# Overcoming Ion Trapping in Chevrel Phase Compounds via Tailored Anion Substitution: An Integrated Study of Theory, Synthesis, and In Operando Techniques for Reversible Aqueous Zn-Ion Batteries

**DOI:** 10.1021/acsami.4c09145

**Published:** 2024-09-13

**Authors:** Yuanshen Wang, Katharina Helmbrecht, Weihao Li, Manuel Dillenz, Yejun Wang, Axel Groß, Alexey Y. Ganin

**Affiliations:** † School of Chemistry, University of Glasgow, G12 8QQ Glasgow, U.K.; ‡ Institute of Theoretical Chemistry, Ulm University, 89081 Ulm, Germany; § Helmholtz Institute Ulm (HIU) for Electrochemical Energy Storage, 89081 Ulm, Germany

**Keywords:** zinc-ion battery, computational materials design, solid-state solutions, in operando battery characterization, powder X-ray diffraction

## Abstract

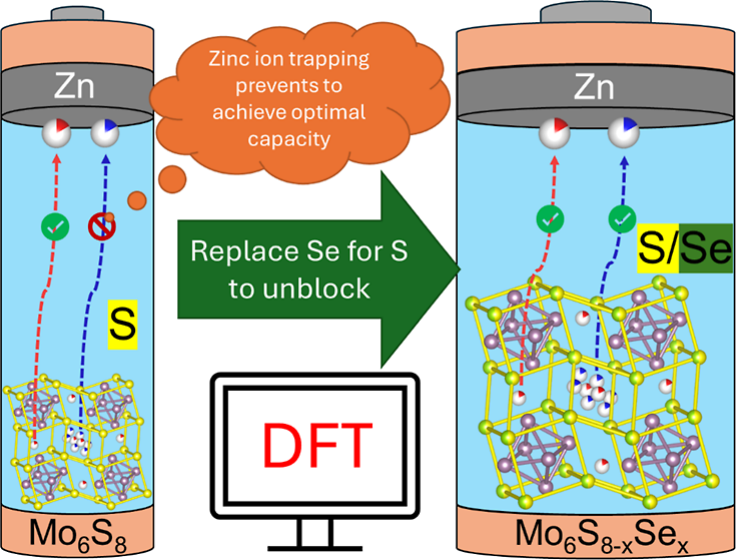

Sustainable batteries are key for powering electronic devices of the future, with aqueous zinc-ion batteries (AZIBs) standing out for their use of abundant, readily available elements, and safer production processes. Among the various electrode materials studied for AZIBs, the Chevrel Phase, Mo_6_S_8_ has shown promise due to its open framework, but issues with zinc ion trapping have limited its practical application. In this work, we employed computational methods to investigate the insertion-deinsertion mechanism in a series of isostructural Mo_6_S_8–*x*_Se_*x*_ (*x* = 0–8) solid solutions as materials that could balance the gravimetric capacity and reversible cycling for AZIBs. Density functional theory (DFT) calculations revealed that increasing the Se content would reduce the binding energy of Zn within the structures, enabling Zn deinsertion compared to the Mo_6_S_8_ structure. Experiments confirmed the formation of Mo_6_S_8–*x*_Se_*x*_ (*x* = 0–8) solid solutions, and electrochemical testing showed improved reversibility of Zn insertion/deinsertion as the amount of Se increased, consistent with the computational predictions. Furthermore, combined in operando X-ray diffraction and electrochemical studies revealed a continuous, gradual Zn-insertion process into Mo_6_S_4_Se_4_, in stark contrast to the abrupt phase changes observed upon Zn insertion in Mo_6_S_8_ and Mo_6_Se_8_. DFT calculations attributed the stabilization of Zn_0.5_Mo_6_S_4_Se_4_ as a prime reason for preventing phase separation, making Se-substituted compounds promising materials for high-performance AZIBs. Overall, this interdisciplinary approach, integrating computational modeling, materials synthesis, and advanced characterization techniques, offers a pathway for fine-tuning anion chemistry that can help create high-performance electrode materials for sustainable energy storage technologies.

## Introduction

Sustainable batteries are key for powering the electronic devices of the future. Aqueous zinc-ion batteries (AZIBs) are particularly promising for this role as they are made from abundant and readily available elements.^1^ Furthermore, as none of the components are significantly moisture or air sensitive, their production can be safer and cheaper than, for example, that of lithium-ion batteries (LIBs).^2,3^ Moreover, in AZIBs zinc metal is utilized directly as the anode material, enabling a high specific volumetric capacity that makes them well-suited for portable and mobile devices where a high energy density in a limited space is required. However, despite significant progress over the past decade, the research in AZIBs is still in its infancy compared with the advances in more established LIB systems.

There is a range of electrode materials studied for the application of AZIBs. Among those many studied to date, a particularly curious case stands out. Mo_6_S_8_ (which is often referred as the Chevrel phase) has a crystal structure that features a highly open framework.^4^ The framework enables short ion transporting pathways and makes Mo_6_S_8_ a promising material for AZIBs.^5^ Consequently, recent work demonstrated that in aqueous electrolyte, the Mo_6_S_8_ cathode reached a very reasonable capacity of 134 mA h g^–1^ on the first discharge.^6^ The cathode showed two discharge plateaus at around 0.5 and 0.35 V due to electrochemical insertion of Zn^2+^ into Mo_6_S_8_ upon formation of ZnMo_6_S_8_ and Zn_2_Mo_6_S_8_. However, there was a significant capacity loss upon further cycling. This was due to zinc-ion trapping, which prevented deinsertion from proceeding beyond ZnMo_6_S_8_ to fully deinserted Mo_6_S_8_. Similar electrochemical behavior, resulting in a significant capacity loss (and confirming that it was impossible to deinsert ZnMo_6_S_8_ fully back to the original Mo_6_S_8_) was reported by other researchers.^7^ This loss of capacity makes Mo_6_S_8_ an unattractive material for AZIBs.

On the other hand, in their recent work Levi et al. have shown that Mg^2+^ ion trapping does not occur within the parental Mo_6_Se_8_ compound (which is isostructural with Mo_6_S_8_).^8^ The trapping was avoided because of the higher polarizability of Se^2–^ anions within the crystal structure of Mo_6_Se_8_. A recent theoretical study elucidated the role of different charge carriers in AZIBs and revealed that a direct relation between the ionic radii and the diffusion barriers of Zn and Mg within the Chevrel phase causes both ions to act similarly in Mo_6_Se_8_.^9^ Indeed, the very recent work showcased the reversibility of Zn^2+^ insertion/deinsertion into Mo_6_Se_8_, leading to a fully reversible AZIB.^10^ Remarkably, the authors suggested that the highly reversible nature of the insertion process into Mo_6_Se_8_ was due to the fact that within the crystal structure, the isolated {Mo_6_} metal clusters remained redox-inert upon the electrochemical process of Zn-ion insertion. Thus, the electrochemical process proceeds without significant altering of the metal bonding and hence preserves the crystal structure integrity, suggesting an opportunity for a highly stable battery. While Mo_6_Se_8_ shows reversibility for Zn insertion/deinsertion, it may not be an optimal electrode material for AZIBs due to its lower theoretical gravimetric capacity compared to Mo_6_S_8_. Tuning the sulfur-to-selenium ratio through rational design of the material’s composition and structure could allow balancing the gravimetric capacity and reversible cycling.

In this work, we employed computational methods to understand the mechanism of insertion in a series of solid-state solutions between Mo_6_S_8_ and Mo_6_Se_8_. Building on our previous studies performed on the Chevrel phases, the research revealed that increasing the Se content within Mo_6_S_8–*x*_Se_*x*_ (*x* = 0–8) solid solutions would reduce the binding energy of Zn within their structures, enabling Zn deinsertion compared to Mo_6_S_8_.^9^ Complementary experimental work and characterization confirmed the formation of Mo_6_S_8–*x*_Se_*x*_ solid solutions. Electrochemical testing showed that reversibility of Zn insertion/deinsertion improves within the solid solutions as the amount of Se increases consistent with density functional theory (DFT) computations. In operando X-ray diffraction studies showed that Mo_6_S_4_Se_4_ exhibits a gradual shift of the key peak positions (rather than abrupt phase changes observed in Mo_6_S_8_ and Mo_6_Se_8_), suggesting that Mo_6_S_4_Se_4_ behaves differently electrochemically from the end members in this solid solution series. These findings demonstrate the importance of tailoring the anion chemistry within Chevrel compounds for achieving efficient multivalent ion insertion in AZIBs.

## Experimental Methods

### Synthesis of Mo_6_S_8–*x*_Se_*x*_ (*x* = 0, 2, 4, 6, and 8)

The targeted compounds were prepared by deinsertion of Cu from the corresponding Cu_2_Mo_6_S_8–*x*_Se_*x*_ (*x* = 0, 2, 4, 6, and 8) powders which were prepared as described in detail in Supporting Information Note 1.^6^ In a typical reaction, 300 mg of Cu_2_Mo_6_S_8–*x*_Se_*x*_ (*x* = 0, 2, 4, 6, and 8) powder was added to 30 mL of 0.5 M Fe(NO_3_)_3_ solution and stirred at 400 rpm for 24 h. The resulting powder was isolated by filtration under vacuum, washed with copious amounts of distilled water, and finally dried at 60 °C overnight. All handling was carried out under ambient conditions without recourse to a protective atmosphere.

### Electrochemical Measurements

Cu_2_Mo_6_S_8–*x*_Se_*x*_, Super P carbon black (CB, Alfa Aesar, ≥99%), and polyvinylidene difluoride (PVDF, Aldrich) were mixed in a weight ratio of 7:2:1 (total mass 50 mg) and ground with a pestle and mortar, followed by the addition of 240 μL of *N*-methyl-2-pyrrolidone (NMP, Sigma-Aldrich, ≥99%). The mixture was ground thoroughly until a uniform black slurry was formed. The slurry was coated onto aluminum foil using a 20 μm coater and dried at 60 °C overnight. Disks with an area of ca. 1.057 cm^2^ (diameter of 1.16 cm) were punched from the foil corresponding to the average loading of the mixture on the foil at 1.14 mg·cm^–2^ (corresponding to 0.798 mg·cm^–2^ of the active Chevrel phase material). A zinc foil (Merck, 99.9%) disk with an area of ca. 1.057 cm^2^ (diameter 1.16 cm, thickness 0.25 cm) served as an anode.

Battery tests were carried out in two types of battery cells at ambient temperature of ca. 18 °C. Swagelok cells were routinely used for all experiments except for in operando XRD studies, as discussed below. The cells were assembled by sandwiching a glass fiber membrane (Whatman GF/A, diameter 13.94 mm, thickness 0.26 mm) wetted with 60 μL of 1 M ZnSO_4_ aqueous solution between two electrodes. Cells were assembled following the order of anode shell, spring, current collector, zinc disk anode, separator, soaking electrolyte, cathode disk, and then cathode shell. For the in operando XRD studies, the CR2032 type cell with the identical cathode, separator, and Zn-metal anode was used but with a window made from Kapton on the cathode side to allow the beam transmittance. Cyclic voltammetry (CV) and galvanostatic charge–discharge (GCD) techniques were conducted on a Biologic, SP-150 (EC-laboratories) potentiostat and a Lanhe battery cycler, respectively.

### Powder X-ray Diffraction Studies

Powder samples were measured on a Rigaku MiniFlex 6G diffractometer (Cu K_α1_ and Cu K_α2_ wavelengths, 1.5406 and 1.5444 Å, respectively) equipped with a D/teX Ultra detector and operating in the Bragg–Brentano geometry. Powder samples were carefully packed onto zero background holders and leveled using a glass microscope slide. The sample holder was spun at a rate of 10 rpm during the measurement. The patterns were collected at a step size of 0.015° and scan rate of 3°/min. In operando X-ray diffraction studies coupled with battery testing were carried out on a Malvern PANalytical Empyrean operating in the Bragg–Brentano geometry using an XRD holder (Malvern PANanlytical, Cu K_α1_ and Cu K_α2_ wavelengths, 1.5406 and 1.5444 Å, respectively) that was designed for a CR2032 coin cell. The diffraction patterns were collected with a step size of 0.013° and scan rate of 4°/min at a repeated scan mode without spin. The crystal structures were refined using GSAS-II software by the Rietveld method.

### Microscopy Studies

For scanning electron microscopy/energy-dispersive X-ray (SEM/EDX) studies, a small amount of sample powder was attached to sticky carbon tape, which was shaken to remove excess powder. The tape was attached to an Al holder. Morphology studies were carried out on a scanning electron microscope (TESCAN CLARA) equipped with a Field Emission Gun electron source, which was coupled with an Oxford Instruments UltimMax 65 with an Aztec live interface EDX system for elemental analysis. The particle size distribution has been evaluated using Nanomeasurer software (Informer Technologies), with ∼200 particles used for the evaluation of each sample.

### Theoretical Calculations

The properties of the materials as well as the ion migration of Zn inside the phase were studied using periodic DFT,^11,12^ as implemented in the Vienna ab initio simulation package.^13−15^ The generalized gradient approximation in the formulation of Perdew, Burke, and Ernzerhof (PBE) was used,^16^ as has been carried out in many DFT studies addressing properties of the Chevrel phase before,^9,17^ as well as the revised PBE functional in combination with the Grimme D3 correction applied.^18,19^ It has also been shown that using the revised PBE functional with a Grimme correction for dispersion forces gives superior results in some cases,^9^ and this approach was thus used for some calculations, which are specified in the main text. Calculations were first performed for the solid solution unit cell of the Chevrel phase with the Brillouin zone sampled using an 8 × 8 × 8 *k*-point grid. For insertion and ion diffusion, different supercells (which are specified in the respective sections) were used, and the *k*-point grid was scaled accordingly in each lattice direction. The electronic structure was converged to 1 × 10^–4^ eV, with a plane-wave cutoff energy of 450 eV.

## Results and Discussion

### Computational Work

**Figure 1:**
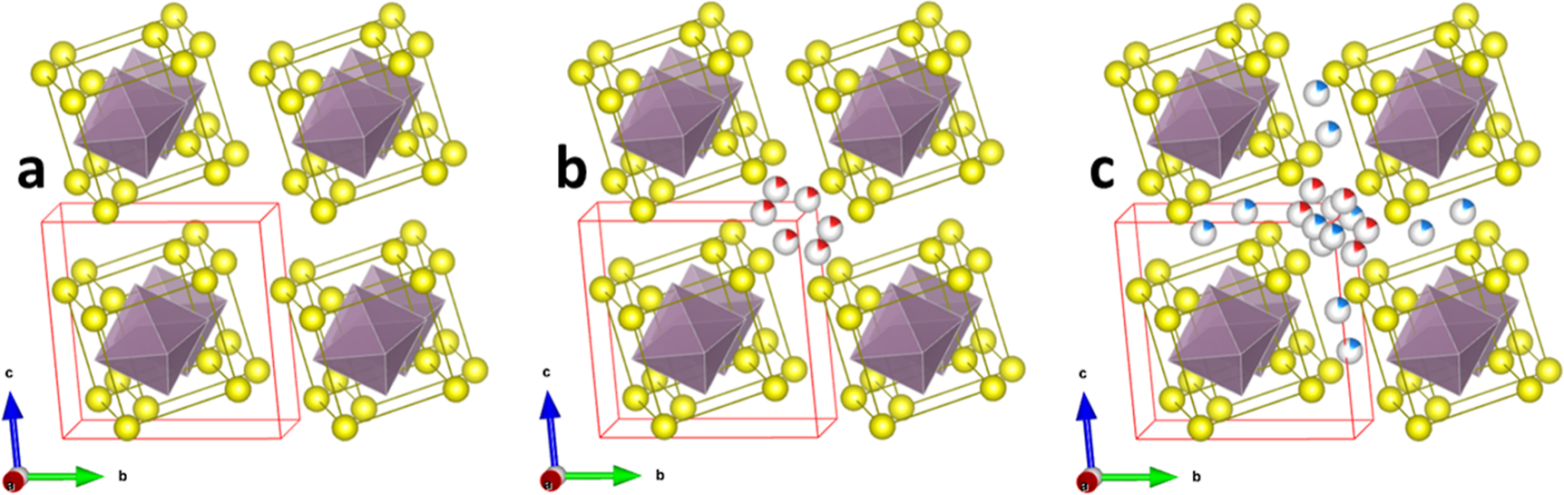
Crystal structures of (a) Mo_6_S_8_, (b) ZnMo_6_S_8_, and (c) Zn_2_Mo_6_S_8_ consisting of Mo_6_S_8_ blocks with Zn-ions taking the cavity positions between them. Mo—magenta, S—yellow, Zn1—gray/red, and Zn2—gray/blue. The unit cell is displayed in red solid line.

As first-principles calculations have been shown to be a viable tool to derive battery properties before,^20^ we took a computational approach to identify the theoretical battery performance. Mo_6_S_8_ and Mo_6_Se_8_ are isostructural compounds and form a series of homogeneous solid solutions.^21^ Therefore, to build Mo_6_S_8–*x*_Se_*x*_ models, we used the Mo_6_S_8_ structure as a basis, incrementally exchanging S with Se. The potential sites for the insertion of Zn ions within the Mo_6_S_8_ structure are shown in Figure 1. There are two different sites within the structure that are available for the insertion of Zn ions yielding the chemical formulas ZnMo_6_S_8_ and Zn_2_Mo_6_S_8_, respectively.

Notably, there are two insertion sites for zinc in Mo_6_S_8_, and each site forms a ring. The first one is the more stable inner ring, while the second site is the less stable outer ring. Within each of these rings, the zinc sites are partly occupied. Each site is only partly occupied to 1/6, creating a significant challenge in describing of structural model using DFT. There are numerous configurations with regard to each of these six partially occupied sites. In addition, there are 720 additional configurations within the combined two-zinc position unit cell, making calculations extremely challenging in terms of computation.

To address this challenge, we followed a previously described approach^9^ and adopted a reduced unit cell (Figures S1–S3) that is still representative of the original crystal structure (Figure 1) since the chemical environment of all atoms is preserved. Due to the equivalency of Zn ions within “the rings” (as discussed at length in our previous paper),^9^ specific positions of the zinc ions can be chosen within the inner and outer rings, respectively. Therefore, it is possible to define the position of Mo and S solely with respect to the Zn1 site and the [Mo_6_S_8_] cluster (Figure S2). Similarly, in Zn_2_Mo_6_S_8_, the [Mo_6_S_8_] cluster and Zn1 site remain, while an additional Zn2 is added (Figure S3).

The reduced cell allowed us to build a range of possible crystal structures, describing numerous configurations within Mo_6_S_8–*x*_Se_*x*_ solid solutions. Consequently, depending on the Se content, the number of the possible configurations can be resolved through a combinatorial approach (Table S1). The number of possible configurations for a given composition within Mo_6_S_8–*x*_Se_*x*_ (*x* = 1–8) depends on the S/Se ratio. For example, in the case of Mo_6_S_7_Se_1_, there are eight different configurations, in the case of Mo_6_S_6_Se_2_, there are 28 possible variations, and so on.

The optimization for the most stable Mo_6_S_8–*x*_Se_*x*_ configurations of the reduced unit cell were carried out using the revised PBE functional in combination with the Grimme D3 correction (RPBE + D3) functional (see Supporting Information Note 1 for more details). The most stable configurations (for a given Se content) are displayed in Figures S4–S10, while their calculated unit cell parameters are summarized in Table S2. The parameters follow a linear trend, which is expected for the formation of solid solutions.

**Figure 2:**
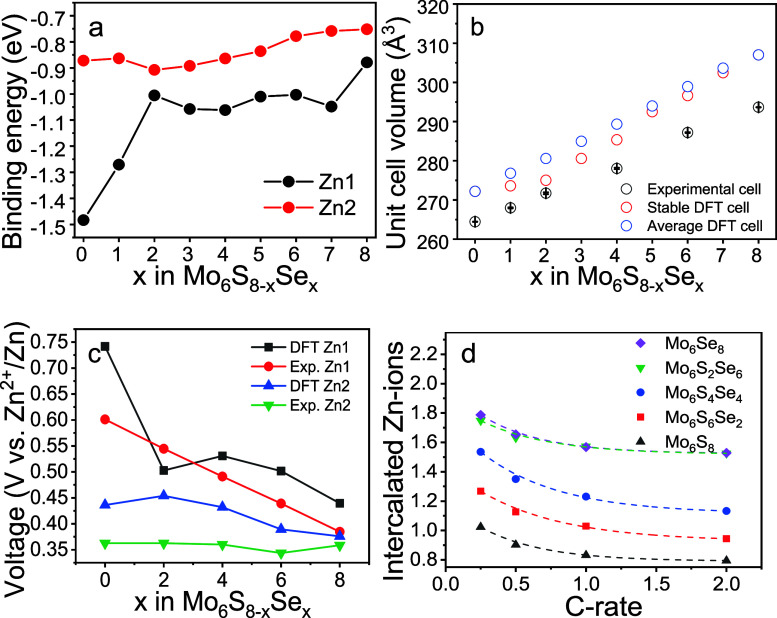
(a) Binding energy of Zn1 and Zn2 in the most stable configuration across Mo_6_S_8–*x*_Se_*x*_ solid solutions as a function of the Se content. (b) Comparison of the unit cell volumes (normalized to the reduced primitive cell) of Mo_6_S_8–*x*_Se_*x*_ from the Rietveld refinement and DFT calculations. (c) Insertion voltage of Zn1 and Zn2 into Mo_6_S_8–*x*_Se_*x*_ structures calculated by DFT and determined experimentally. (d) Rate performance of Mo_6_S_8–*x*_Se_*x*_ at 0.25, 0.5, 1, and 2 C, the number of inserted Zn was calculated from the third cycle’s data.

The binding energies of Zn1 and Zn2 ions within the most stable configurations are plotted in Figure 2a as a function of Se content.

In addition, Figure S11 shows binding energies for all possible configurations of the crystal structures. It should be noted that the binding energies in Figure 2a are derived with RPBE + D3, while all PBE values show the same trend (Figure S11). In previous research,^9^ PBE provides the closest lattice parameters than RPBE + D3, but underestimate the energy (as well as insertion voltages), where RPBE + D3 was opposite. However, the difference in the specific value will not affect the energy/lattice parameters/insertion voltage trends in Mo_6_S_8–*x*_Se_*x*_ solid solution systems. Since Mo_6_S_8_ and Mo_6_Se_8_ were selected as references, the consistency of the trend of the solid solution energy change in the PBE function and the RPBE + D3 function proves that there are no unconsidered parameters in the system.

Previous experimental data showed that during the initial electrochemical process two Zn ions would insert into Mo_6_S_8_ upon formation of Zn_2_Mo_6_S_8_.^6,7^ However, upon discharge, only one Zn can be deinserted from the Zn_2_Mo_6_S_8_. Hence, the deinsertion does not proceed beyond ZnMo_6_S_8_, preventing the reversibility of Mo_6_S_8_ and dramatically reducing the battery capacity. The inability of Zn to deinsert from ZnMo_6_S_8_ is due to a large binding energy of Zn1 ions within the Mo_6_S_8_ structure (Figure 2a). However, as S is gradually replaced with Se in Mo_6_S_8–*x*_Se_*x*_, the binding energy of Zn1 is reduced drastically. In particular, the binding energy of Zn1 drops sharply from −1.48 eV in Mo_6_S_8_ to −1.01 eV in Mo_6_S_6_Se_2_ and stays consistent around −1.00 eV for the remaining solid solutions until finally reaching −0.88 eV for Mo_6_Se_8_. This suggests that replacing S with Se would lead to a more facile deinsertion of Zn1. Finally, a closer examination of the binding energies (Figure S11) reveals that the location of the Se sites within the reduced unit cell plays a role, but it is evident that the Zn binding energy follows a downward trend consistent with the replacement of S by Se.

### Synthesis and Characterization of Mo_6_S_8–*x*_Se_*x*_ (*x* = 0, 2, 4, 6, and 8)

Since DFT calculations predicted that replacing S with Se in Mo_6_S_8_ would lead to an improved deinsertion process, five Mo_6_S_8–*x*_Se_*x*_ solid solutions with *x* = 0, 2, 4, 6, and 8 were synthesized (see Supporting Information Note 1 and Experimental Methods). The Rietveld refinement of the crystal structure model based on Mo_6_S_8_ (space group: *R*3̅*m*)^22^ against the experimental PXRD data (Figures S12–S16) showed a good match between the experimental and calculated profiles. In the case of Mo_6_Se_8_, the final profile (Figure S16) revealed some unfitted peaks which were consistent with the presence of a small impurity of hexagonal MoSe_2_.^23^ The refined unit cell volumes of the experimentally prepared phases showed a near linear trend confirming the formation of a series of solid solutions in line with Vegard’s law (Figure 2b). The structural parameters obtained from the Rietveld refinement for Mo_6_S_8–*x*_Se_*x*_ are summarized in Table S3. There is good agreement with the previously reported crystal data in the literature especially for the two end members of the series: Mo_6_S_8_ and Mo_6_Se_8_.^6,24,25^ However, due to the expected random distribution of S and Se within the relevant Mo_6_S_*x*–8_Se_*x*_ crystal structure, the experimental unit cell volumes deviate from the most stable energy configurations obtained by DFT (which assumed the fixed position of the S or Se within the cell). However, when the average unit cell volumes are considered over all configurations, the linear trend of the calculated volumes is restored and remains consistent with the experimental unit cell volumes (Figure 2b). The powder diffraction patterns of the synthesized samples (Figure S17) showed a gradual shift of the reflections toward lower angles which is consistent with the increased unit cell volume and the replacement of S with Se within the crystal structure.

As the difference in particle sizes between the samples can cause variability in the electrochemical performance,^26^ the particle size distributions across Mo_6_S_8–*x*_Se_*x*_ (*x* = 0, 2, 4, 6, and 8) were evaluated by SEM microscopy (Figure S18). There is significant variation in particle sizes within each sample, ranging from 0.5 to 4.8 μm. However, across all prepared samples, the morphology remains relatively consistent, as evidenced by average particle sizes ranging between 1.43 and 1.63 μm (Figure S19). This ensures that the electrochemical performance across the samples is not influenced by the particle sizes. Finally, the EDX (with representative spectra displayed in Figure S20) confirmed that the samples were fully deinserted and confirmed the absence of peaks associated with Cu or Fe. The compositions of the experimental samples were close to the expected ones as well confirming a good agreement with the expected ratios (Table S4).

### Zn-Ion Battery Performance of Mo_6_S_8–*x*_Se_*x*_ (*x* = 0, 2, 4, 6, and 8)

**Figure 3:**
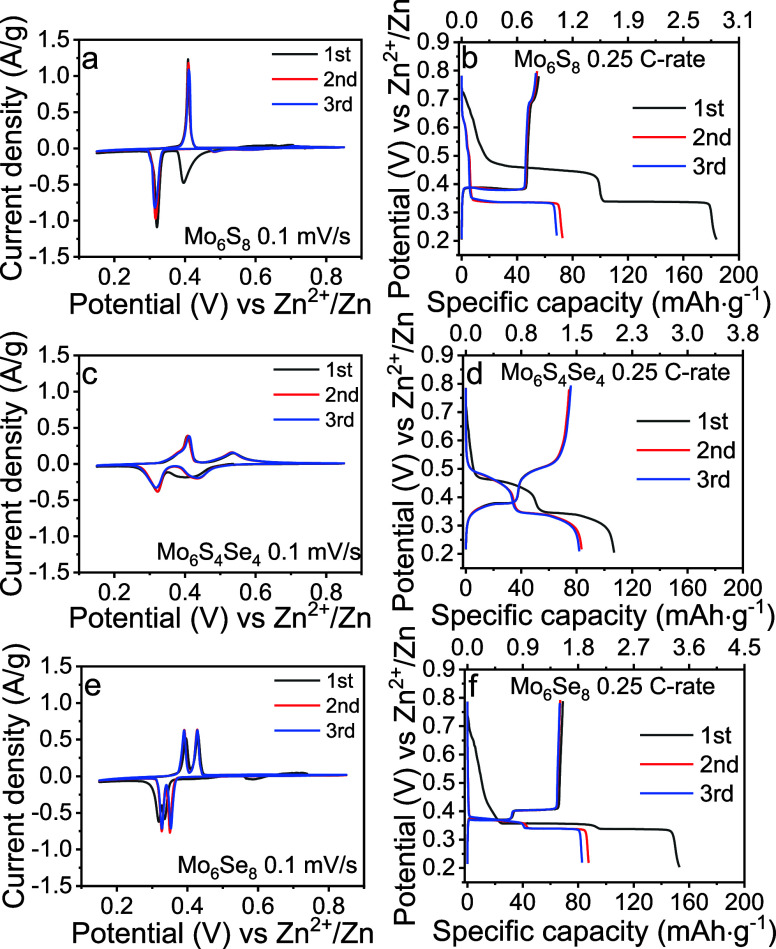
CV curves recorded at 0.1 mV/s and GCD profiles at 0.25 *C*-rate of (a,b) Mo_6_S_8_, (c,d) Mo_6_S_4_Se_4_, and (e,f) Mo_6_Se_8_. All measurements started from OCV for the initial three cycles.

A series of CV experiments were carried out (Figures 3 and S21, S22) to probe the effect of the S substitution for Se within Mo_6_S_8–*x*_Se_*x*_. During the first negative scan, Mo_6_S_8_ displays two distinctive cathodic peaks at ∼0.32 and ∼0.40 V vs Zn^2+^/Zn corresponding to the insertion of two Zn ions upon formation of ZnMo_6_S_8_ and Zn_2_Mo_6_S_8_, respectively. However, upon the reverse scan, only the peak at ∼0.40 V remains, suggesting that the deinsertion of Zn from ZnMo_6_S_8_ is impeded. These observations for Mo_6_S_8_ are consistent with the previous research.^6^

Notably, the substitution of S with Se enables the Zn deinsertion process, as evident from the presence of two peaks in the cycling voltammetry data. For example, the close analysis of the CV data suggests that the insertion into Mo_6_S_4_Se_4_ (Figure 3c) and Mo_6_S_2_Se_6_ is fully reversible (Figure S22). However, there is a distinctive broadening of the peaks of the CV scans of these compounds, suggesting a difference in the insertion process compared to those of Mo_6_S_8_ (Figure 3a) and Mo_6_Se_8_ (Figure 3e) as discussed later. Furthermore, within the Mo_6_S_8–*x*_Se_*x*_ (*x* = 2; 4; 6) series, the peaks associated with the insertion of Zn1 tend to appear at significantly higher voltages than that in the end members of the series. However, the theoretical voltages for the insertion of Zn1 and Zn2 into the relevant Mo_6_S_8–*x*_Se_*x*_ compounds are consistent with the experimental values (Figure 2c), suggesting that the observed behavior is expected for the solid solution series. The experimental insertion voltages for Zn1 decrease as the Se-to-S ratio increases (showing a trend similar to the DFT results), while the voltage values for Zn2 remain broadly unchanged irrespective of the Se content. In this regard, Mo_6_S_2_Se_6_ and Mo_6_Se_8_ show excellent reversibility in line with the DFT calculations of the binding energy (Figure 2a) which predicted that substituting S with Se would enable a fully reversible process down to fully deinserted chalcogenides.

The galvanostatic charge–discharge (GCD) curves of Mo_6_S_8–*x*_Se_*x*_ (*x* = 0, 2, 4, 6, and 8) were recorded at 0.25 C (Figures 3 and S21, S22) and 1 C (Figure S23) rates to study the effect of the substitution on the specific capacity of the battery with the relevant data summarized in Table S5. Notably, taking the third cycle as a benchmark, the highest capacity (at rates of 1 and 2 C relevant to AZIB applications) is achieved by Mo_6_S_2_Se_6_ rather than Mo_6_Se_8_. The same number of Zn is inserted into Mo_6_S_2_Se_6_ as into Mo_6_Se_8_ (Figure 2d), suggesting that solid solution compounds are the way forward for reaching the optimal capacity.

Furthermore, consistent with the CV data, the GCD curves of Mo_6_S_8_ (Figure 3b) and Mo_6_Se_8_ (Figure 3f) exhibited flat plateaus at voltages similar to those in the CV scans and without obvious potential drops. However, for the three partially substituted Chevrel phases, the curves demonstrated a sloping form with the voltages continuing to increase/decrease along the whole charge/discharge stages, consistent with the broad peaks in the CV data. To test the possible origin of the slopes in GCD, we carried out in operando data studies on Mo_6_S_8_, Mo_6_S_4_Se_4_, and Mo_6_Se_8_.

### In Operando Studies of Zn-Ion Battery Performance of Mo_6_S_8_, Mo_6_S_4_Se_4_, and Mo_6_Se_8_

**Figure 4:**
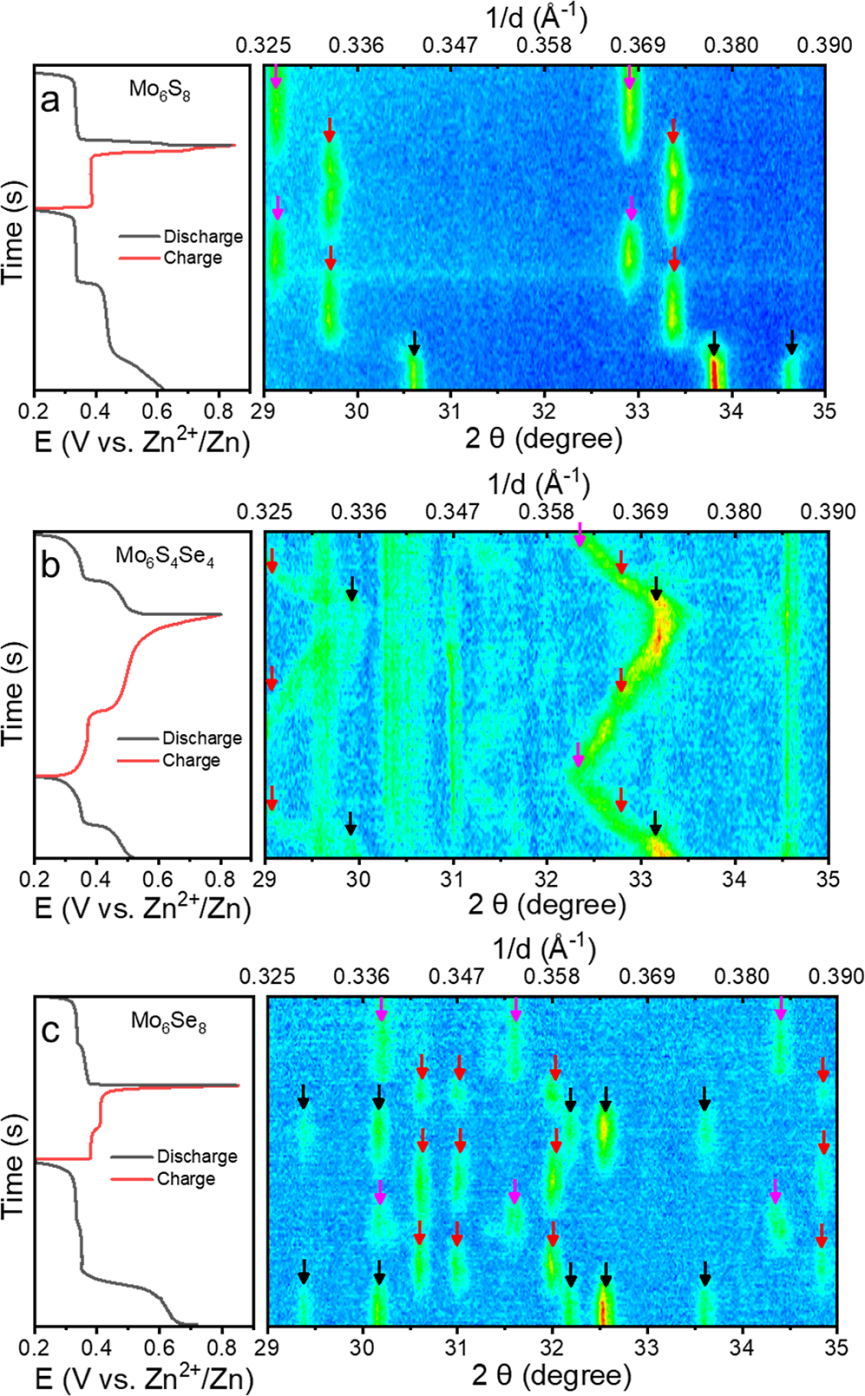
Contour plots of in operando XRD patterns and the accompanying charge/discharge curves (collected at 0.2 *C*-rate) of (a) Mo_6_S_8_, (b) Mo_6_S_4_Se_4_, and (c) Mo_6_Se_8_. Black arrows mark the peaks of the pristine (uninserted) phases, and red and magenta arrows mark the peaks of the inserted phases with one zinc and two zinc ions, respectively.

The phase evolution of Zn insertion/deinsertion into Mo_6_S_8_ has been investigated by Chae et al.,^6^ however, only using ex situ PXRD experiments. Therefore, to understand the variation of electrochemical performance with the increasing Se ratio, we carried out in operando PXRD studies combined with simultaneous GCD tests on Mo_6_S_8_, Mo_6_S_4_Se_4_, and Mo_6_Se_8_ to examine the effect of electrochemistry on phase composition (Figure 4).

The measurements were carried out within a limited 2θ range to specifically target the most significant peaks pertinent to Mo_6_S_8_, Mo_6_S_4_Se_4_, and Mo_6_Se_8_. In line with the electrochemical data, during the initial discharge plateau, the peaks associated with Mo_6_S_8_ disappear, indicative of the Zn1 insertion and the appearance of the ZnMo_6_S_8_ peaks (Figure 4a). Subsequently, the second plateau is consistent with the formation of the Zn_2_Mo_6_S_8_ phase, as evident from the X-ray diffraction. However, upon follow-up cycles, only peaks related to the ZnMo_6_S_8_ and Zn_2_Mo_6_S_8_ (but not of Mo_6_S_8_) are visible, which is consistent with a partial deinsertion process.

Remarkably, the in operando data for Mo_6_S_4_Se_4_ (Figure 4b) revealed different behavior. There is a gradual shift in the PXRD reflection positions upon cycling which suggests that a series of Zn_2–*y*_Mo_6_S_4_Se_4_ (*y* = 0–2) solid solutions are formed. The unit cell parameters and unit cell volume follow a gradual trend consistent with the formation of solid-state solutions upon Zn insertion (Figure S24). This is in stark contrast to Mo_6_S_8_ where discrete ZnMo_6_S_8_ and Zn_2_Mo_6_S_8_ phases only exist. The formation of a solid solution explains the noticeable gradient in the charge–discharge curves for Mo_6_S_4_Se_4_ compared to those observed for Mo_6_S_8_ and Mo_6_Se_8_ (Figure 3).

Contrary to Mo_6_S_8_, the in operando PXRD data show that the insertion of Zn into Mo_6_Se_8_ (Figure 4c) is fully reversible, as evidenced by the peaks of all three Mo_6_Se_8_, ZnMo_6_Se_8_, and Zn_2_Mo_6_Se_8_ phase invariably being present in the diffraction patterns during the electrochemical process. However, these are discrete phases with no intermediate Zn_2–*y*_Mo_6_Se_8_ phase being formed upon insertion. The comparison of unit cell parameters determined for the phase at the maximum insertion level further confirms its consistency with literature findings (Table S6), thus validating the reliability of the in operando experiment conducted in this work.

Thus, in operando experiments confirm the results of the ex situ electrochemical experiments and the results of the DFT calculations that due to significantly lower binding energies of Zn1 ions, it is possible to remove Zn ions fully from ZnMo_6_S_4_Se_4_ and ZnMo_6_Se_8_. However, the striking difference in the insertion process, as evidenced by the distinctive slopes upon the insertion of Mo_6_S_4_Se_4_ to ZnMo_6_S_4_Se_4_ and the nearly horizontal plateaus in the case of the Mo_6_Se_8_ to ZnMo_6_Se_8_ process, requires additional explanation. Therefore, detailed DFT calculations were carried out to explain these phenomena.

### Providing Explanation for a Gradual Insertion into Mo_6_S_4_Se_4_

As noted above, unlike Mo_6_S_8_ and Mo_6_Se_8_, Mo_6_S_4_Se_4_ does not show discrete phases formation upon insertion rather it forms continuous Zn_*y*_Mo_6_S_4_Se_4_ (*y* = 0–1) solid solution phases (Figure 4b). To understand this unusual behavior of Mo_6_S_4_Se_4_, DFT calculations were applied to determine the convex hull for Zn1 insertion into Zn_*y*_Mo_6_S_8_, Zn_*y*_Mo_6_S_4_Se_4_, and Zn_*y*_Mo_6_Se_8_ across a range of *y* = 0–1 (Figure S25). In this framework, the aim was to determine the mixing energy per atom. Values of the mixing energy below the energy hull indicate that an intermediate phase is more stable. For example, in the case of *y* = 0.5, the corresponding mixing energy for Zn_0.5_Mo_6_S_8_ is significantly above the energy hull, suggesting that Zn_0.5_Mo_6_S_8_ is unstable and would be prone to phase separation into Mo_6_S_8_ and Zn_1_Mo_6_S_8_. On the contrary, at *y* = 0.5, the resulting Zn_0.5_Mo_6_S_4_Se_4_ is below the energy hull, and thus, there is no tendency for it to segregate into Zn_1_Mo_6_S_4_Se_4_ and Mo_6_S_4_Se_4_.

The results point out that there is a clear miscibility gap for Zn insertion in the case of Mo_6_S_8_, whereas intermediate Zn concentrations are only slightly unstable for Mo_6_Se_8_ indicating phase separation in agreement with the in operando XRD studies. In contrast, the Mo_6_S_4_Se_4_ shows stable configurations at Zn contents of *y* = 0.5, resulting in a change of the mode of insertion to a solid solution in agreement with the charge–discharge profiles and in operando data.

## Conclusions

In conclusion, the DFT calculations reveal that replacing more than two sulfur atoms with selenium in the Mo_6_S_8–*x*_Se_*x*_ (*x* = 0–8) effectively lowers the binding energy of zinc ions, mitigating the ion trapping issues that have beset the battery performance of the parent Mo_6_S_8_ compound. Notably, the calculations suggest that further increasing the selenium content beyond the *x* = 2 threshold does not significantly enhance the binding energy reduction. Consequently, only a modest selenium substitution level is required to unlock the reversible zinc insertion and deinsertion behavior that is essential for high-performance aqueous zinc-ion battery cathodes. Experimental work confirms these findings with the cycling voltammetry on samples with *x* > 2 demonstrating distinctive peaks suggesting reversibility of Zn-ion insertion/deinsertion. The optimal performance was found for Mo_6_S_2_Se_6_ that outperforms Mo_6_Se_8_ at battery relevant high charging rates. The combined in operando X-ray diffraction and electrochemical studies unveil a remarkable difference in the zinc insertion behavior between two end members (Mo_6_S_8_ and Mo_6_Se_8_) of the series and Mo_6_S_4_Se_4_. Unlike the end members that undergo abrupt phase transitions into ZnMo_6_S_8_ and ZnMo_6_Se_8_ phases upon zinc insertion, Mo_6_S_4_Se_4_ exhibits continuous solid solution behavior, forming Zn_*y*_Mo_6_S_4_Se_4_ (*y* = 0–1) phases without abrupt phase transformation. This unusual characteristic of Mo_6_S_4_Se_4_ was elucidated by DFT calculations that evaluated the mixing energy during zinc insertion. The computational results clearly highlight the existence of a miscibility gap for zinc insertion in Mo_6_S_8_, whereas Mo_6_S_4_Se_4_ exhibits stable crystal structure configurations at zinc contents of *y* = 0.5, facilitating the formation of a continuous phase ranges. This fundamental difference in the insertion mechanism, arising from the tailored anion chemistry, is consistent with the charge–discharge profiles and in operando data. Overall, the synergistic combination of computational modeling and experimental validation provides valuable insights into optimizing the anion framework, paving the way for the development of advanced electrode materials with tailored properties for next-generation AZIBs.

## Supporting Information Available

Information regarding the materials, method of synthesis, and characterization; calculated molecular geometry; binding energy of solid solutions, Rietveld refinement of solid solutions; SEM images; EDXS spectra of solid solutions; battery test details; and mixing energies of solid solutions (PDF)

## Abbreviations

AZIBs: aqueous zinc-ion batteries;
DFT: density functional theory
EDX: energy-dispersive X-ray
LIBs: lithium-ion batteries
CP: Chevrel phase
CV: cyclic voltammetry
GCD: galvanostatic charge–discharge
PBE: Perdew–Burke–Ernzerhof
RPBE + D3: revised PBE functional in combination with the Grimme D3 correction
PXRD: powder X-ray diffraction
SEM: scanning electron microscope
XRD: X-ray diffraction
