# Supplementary material for: Overcoming Ion Trapping in Chevrel Phase Compounds via Tailored Anion Substitution: An Integrated Study of Theory, Synthesis, and In Operando Techniques for Reversible Aqueous Zn-Ion Batteries
Source: ACS Appl Mater Interfaces. 2024 Sep 13;16(38):50671–8. doi: 10.1021/acsami.4c09145 (PMC11440462; doi:10.1021/acsami.4c09145)
Supplement: Supplementary file 1 — am4c09145_si_001.pdf [file am4c09145_si_001.pdf]

Supporting information

Overcoming Ion Trapping in Chevrel Phase Compounds via Tailored Anion Substitution: An Integrated Study of Theory, Synthesis, and In-Operando Techniques for Reversible Aqueous Zn-Ion Batteries

*Yuanshen Wang[1], Katharina Helmbrecht[2], Weihao Li[1], Manuel Dillenz[2], Yejun Wang[1], Axel Groß[2,3]*and Alexey Y. Ganin[1]**

1. School of Chemistry, University of Glasgow, G12 8QQ, Glasgow, United Kingdom

2. Institute of Theoretical Chemistry, Ulm University, 89081, Ulm, Germany

3. Helmholtz Institute Ulm (HIU) for Electrochemical Energy Storage, 89081, Ulm, Germany

Corresponding Authors

Axel Groß: axel.gross@uni-ulm.de

Alexey Y. Ganin: Alexey.Ganin@glasgow.ac.uk

Supplementary Note 1 - Experimental details:

Synthesis of $Cu_2Mo_6S_8$ and $Cu_2Mo_6Se_8$

$Cu_2Mo_6S_8$ was made in advance through traditional solid-state routine.[1] Specifically, stoichiometric element powders of 0.1335 g copper (Cu), 0.5756 g molybdenum (Mo) and 0.2566 g sulfur (S) were added into a quartz ampoule through a long stem funnel without grinding in an argon filled glovebox. To be noticed that stoichiometric amount of copper should be 0.1271 g, however, 5% weight more was added due to very fine powder which stuck on the wall of funnel and caused obvious loss. Then the ampoule was evacuated to lower than 5×10^{-3} mbar and sealed via a propane/oxygen torch subsequently. The mixture in a sealed ampoule was shaken gently to become homogeneous before being placed in the middle of muffle furnace and heated. The calcination process was set as 0.5 °C/min to 400 °C keeping 5 min, then 2 °C/min to 1050 °C keeping 48 h, finally 5 °C/min cooling down to room temperature. The initial ramping rate was incredibly low to prevent rapid sulfur vapor generation which led to the high inner pressure and explosion of ampoule. When the first calcination was finished, the ampoule was taken out and opened via a sharp tube opener, the gained powder was ground using mortar and pestle to homogenize again, resealed and evacuated to similar vacuum value. The reheat process was set as 5 °C/min to 1050 °C keeping 48 h, then 5 °C/min cooling down to room temperature. The final product was ash black powder.

The synthesis routine of $Cu_2Mo_6Se_8$ was quite similar to $Cu_2Mo_6S_8$ formation with slight change in heat process. In particular, stoichiometric element powders of 0.1335 g Cu, 0.5756 g Mo and 0.6317 g selenium lump (Se) were added into a quartz ampoule through a long stem funnel without grinding. Excess 5 % copper was added as well, the evacuation was same as mentioned. The calcination process was set as the same as the $Cu_2Mo_6S_8$ except the slight variation from 400 °C to 685 °C in first achieved temperature, due to the boiling point changing

from S to Se. The subsequent resealing and annealing remained the same. The final product also appeared as powdery ash black substance.

Synthesis of $Cu_2Mo_6S_{8-x}Se_x$ (x = 2, 4, 6)

Instead of using traditional solid-state routine from element powders,[2] a novel and simplified process was applied by heating a mixture of aforehand synthesized two Chevrel phase precursor $Cu_2Mo_6S_8$ and $Cu_2Mo_6Se_8$ at different mixing ratios (400 – 500 mg products were aimed at). The molar ration mixing was depending on the S and Se in $Cu_2Mo_6S_8$ and $Cu_2Mo_6Se_8$ respectively. Mixtures were added into a quartz ampoule through a long stem funnel, evacuated and shaken, then heated: 5 °C/min to 1050 °C keeping 48 h, then 5 °C/min cooling down to room temperature. Reannealing with exactly the same process was generally required.

Calculations of binding energies for the Zn ions

Using the relevant configurations, the binding energies of the zinc ions within $Mo_6S_{8-x}Se_x$ were calculated using the following equation (1):

$$E_{binding} = E(Zn_yMo_6S_{8-x}Se_x) - (E(Zn_{y-1}Mo_6S_{8-x}Se_x) + (y-1)\,E(Zn)) \quad (1)$$

where, y is the Zn-site occupancy composition; $E(Zn_yMo_6S_{8-x}Se_x)$ and $E(Zn_{y-1}Mo_6S_{8-x}Se_x)$ are the total energy of the optimized $Zn_yMo_6S_{8-x}Se_x$ and $Zn_{y-1}Mo_6S_{8-x}Se_x$ and $E(Zn)$ is the total energy of one Zn metal atom from a metal reference.

Similarly, the voltages were calculated using the following equation (2) and (3):

$$V = -1/2\,(E(Zn_yMo_6S_{8-x}Se_x) - E(Zn_{y-1}Mo_6S_{8-x}Se_x) - E(Zn)) \quad (2)$$

$$V = -1/2\,E_{binding} \quad (3)$$

However, using PBE, as previously discussed, the voltages were severely underestimated at values only up to 0.25 V, compared to the experimental value of 0.6 V. Since we would expect theoretical voltages always to be slightly higher than experimental values, due to their ideal charge transfer without hindrance at the SEI or other barriers, a different approach was needed. Previous computational studies of Mo_6S_8 showed that using RPBE+D3 yields higher voltages,[3] slightly overestimating them compared to experiments. Thus, the previously described systems for evaluating the binding energies were recalculated with this functional.

Supplementary Figures

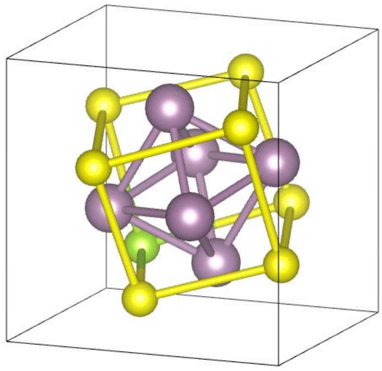

Figure S1. A possible configuration of the reduced unit cell of $Mo_6S_7Se_1$. In the case of $Mo_6S_7Se_1$, there are 8 different variations of how Se is located with the cell. (Mo: Magenta, S/Se: Yellow/green).

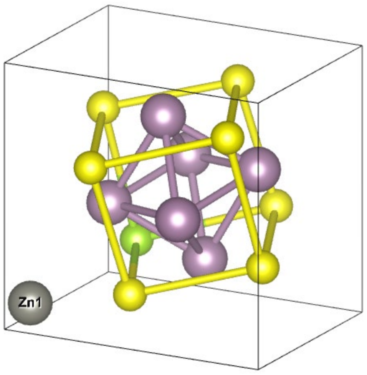

Figure S2. A possible configuration of the reduced unit cell of $ZnMo_6S_7Se_1$. In the case of $ZnMo_6S_7Se_1$, there are 8 different variations of how Se is located with the cell. (Mo: Magenta, S/Se: Yellow/green).

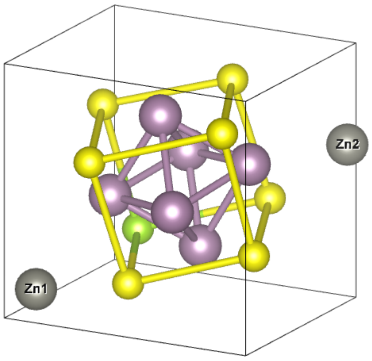

Figure S3. A possible configuration of the reduced unit cell of $Zn_2Mo_6S_7Se_1$. In the case of $Zn_2Mo_6S_7Se_1$, there are 8 different variations of how Se is located with the cell. (Mo: Magenta, S/Se: Yellow/green).

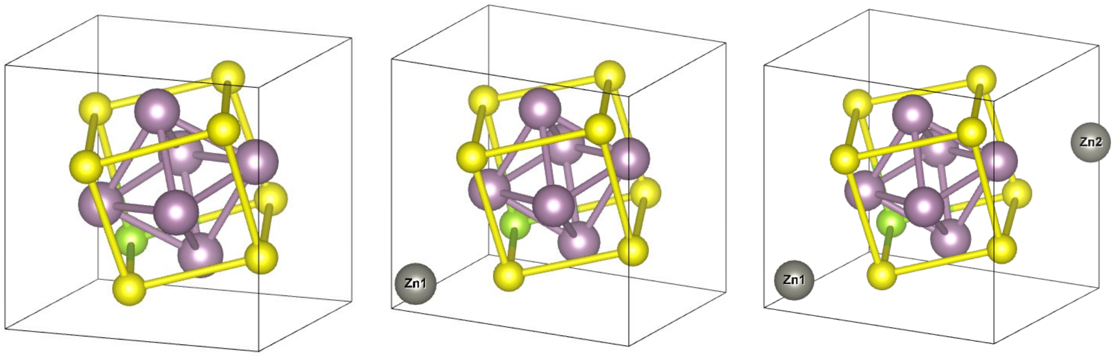

Figure S4. The optimized structures of $Mo_6S_7Se_1$, $ZnMo_6S_7Se_1$ and Zn_2 $Mo_6S_7Se_1$. The configurations depicted represent the most energetically stable structures (Zn: Gray, Mo: Magenta, S/Se: Yellow/green).

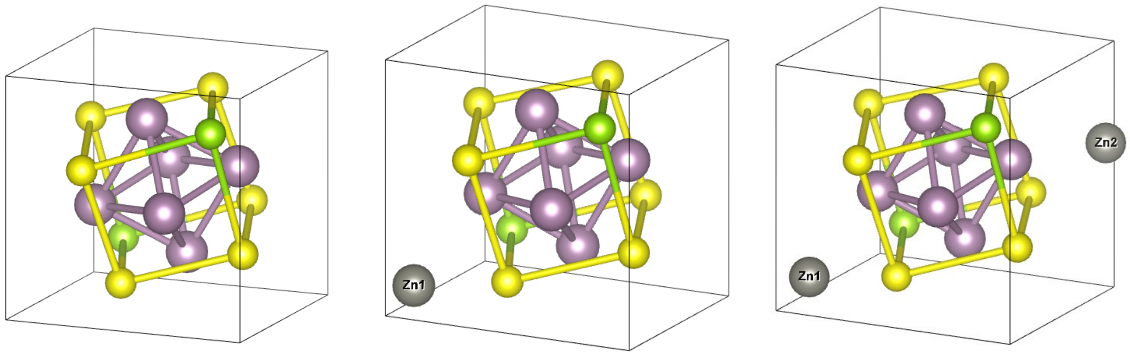

Figure S5. The optimized structure of $Mo_6S_6Se_2$, $ZnMo_6S_6Se_2$ and $Zn_2Mo_6S_6Se_2$. The configurations depicted represent the most energetically stable structures (Zn: Gray, Mo: Magenta, S/Se: Yellow/green).

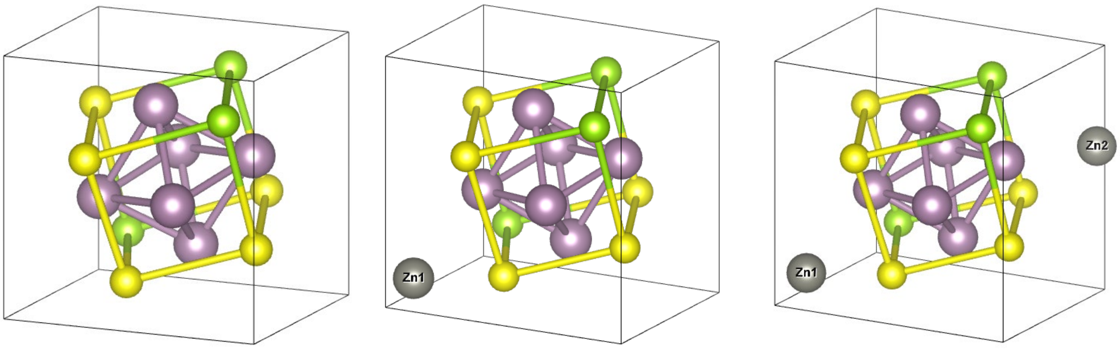

Figure S6. The optimized structure of $Mo_6S_5Se_3$, $ZnMo_6S_5Se_3$ and $Zn_2Mo_6S_5Se_3$. The configurations depicted represent the most energetically stable structures (Zn: Gray, Mo: Magenta, S/Se: Yellow/green).

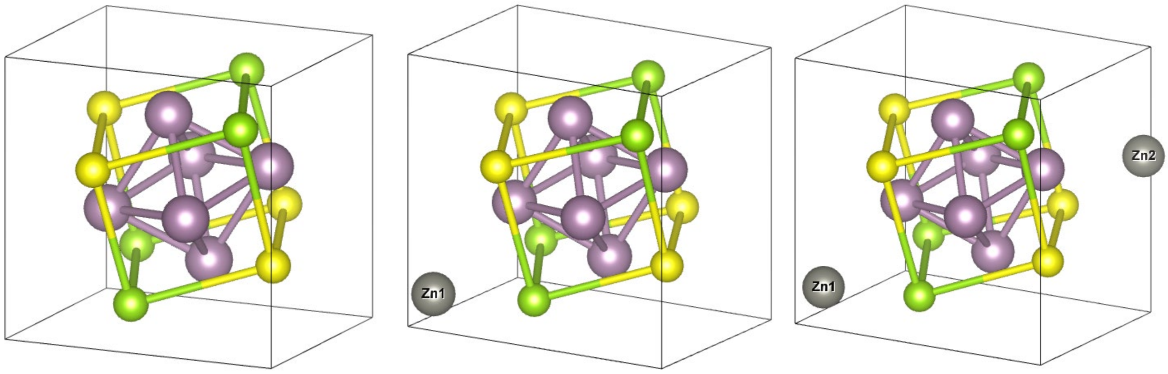

Figure S7. The optimized structure of $Mo_6S_4Se_4$, $ZnMo_6S_4Se_4$ and $Zn_2Mo_6S_4Se_4$. The configurations depicted represent the most energetically stable structures (Zn: Gray, Mo: Magenta, S/Se: Yellow/green).

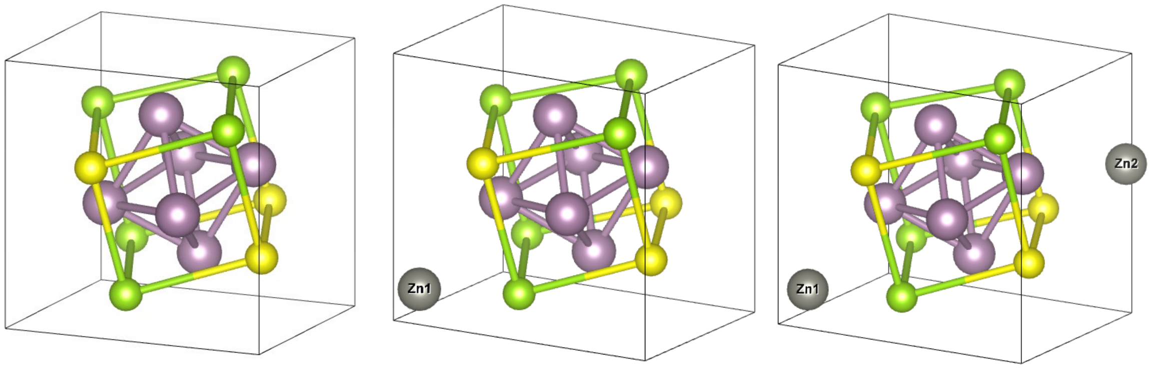

Figure S8. The optimized structure of $Mo_6S_3Se_5$, $ZnMo_6S_3Se_5$ and $Zn_2Mo_6S_3Se_5$. The configurations depicted represent the most energetically stable structures (Zn: Gray, Mo: Magenta, S/Se: Yellow/green).

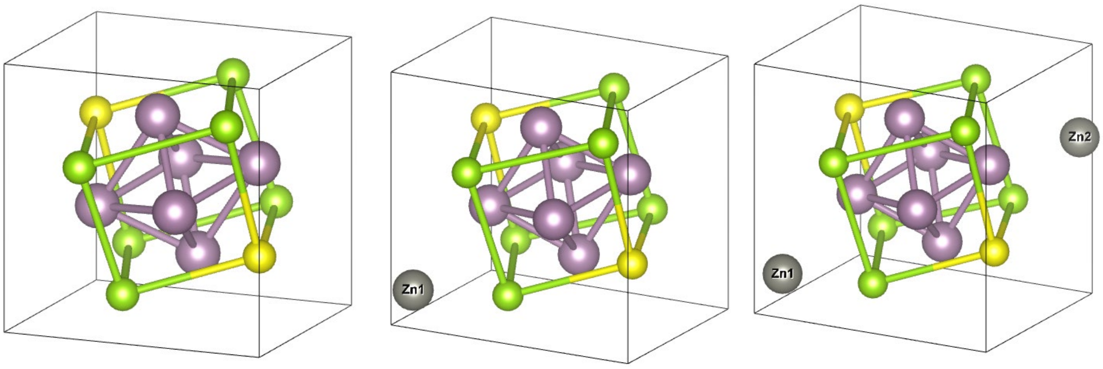

Figure S9. The optimized structure of $Mo_6S_2Se_6$, $ZnMo_6S_2Se_6$ and $Zn_2Mo_6S_2Se_6$. The configurations depicted represent the most energetically stable structures (Zn: Gray, Mo: Magenta, S/Se: Yellow/green).

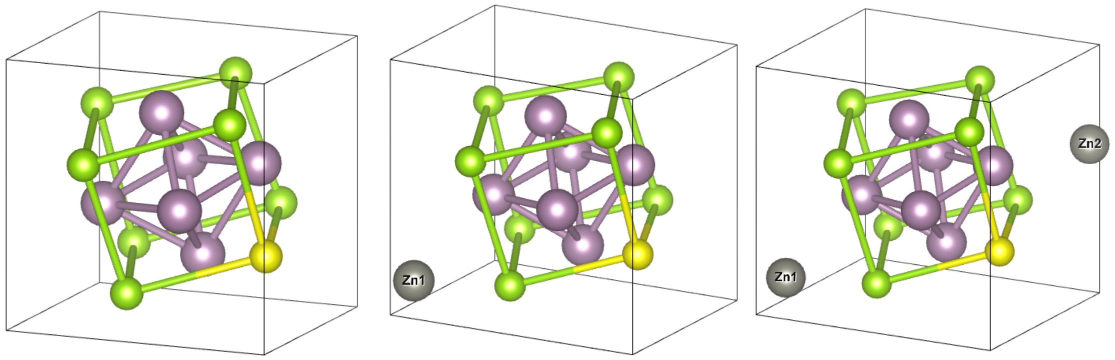

Figure S10. The optimized structure of $Mo_6S_1Se_7$, $ZnMo_6S_1Se_7$ and $Zn_2Mo_6S_1Se_7$. The configurations depicted represent the most energetically stable structures (Zn: Gray, Mo: Magenta, S/Se: Yellow/green).

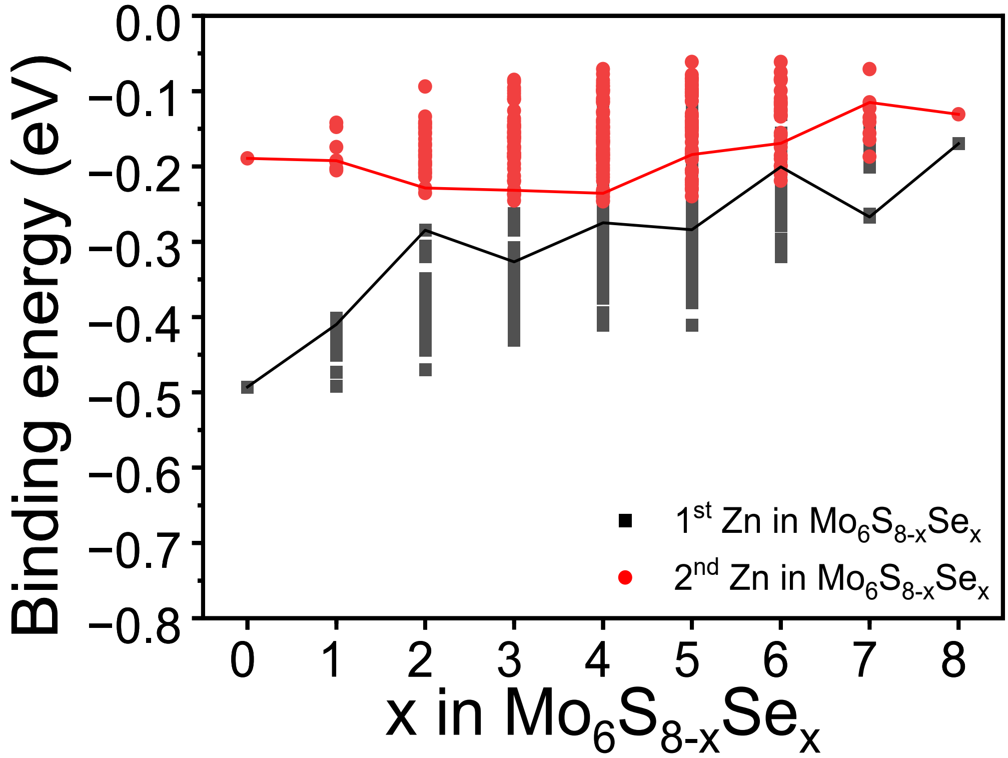

Figure S11. The binding energy of zinc ion within the series of $Mo_6S_{8-x}Se_x$ solid state solutions. The points links by line represent the most stable configuration across Zn(1) and Zn(2) in the $Mo_6S_{8-x}Se_x$ solid solution. Each point exemplifies an individual configuration (whether energetically stable or unstable) of the reduced cell.

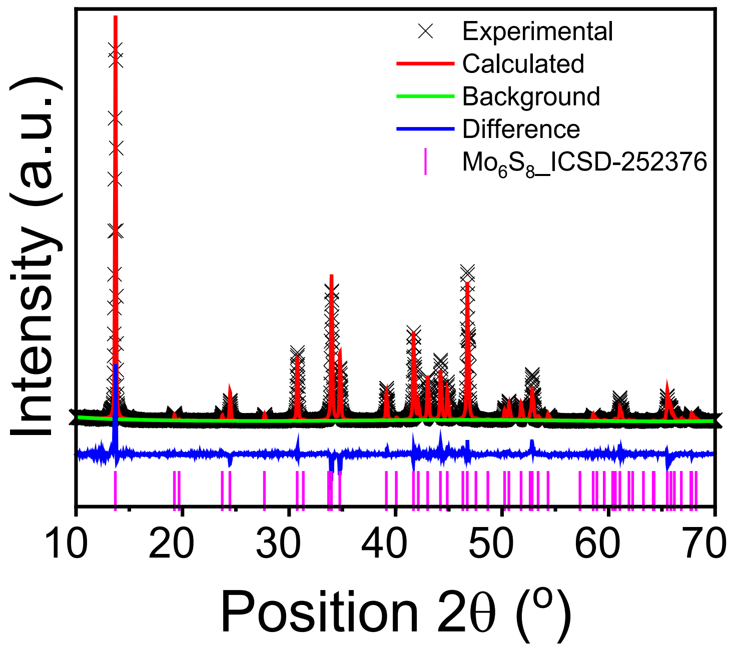

Figure S12. Rietveld refinement of the experimental PXRD profile (CuKα) for a sample with a nominal composition Mo_6S_8 against a structure model (Space group: R-*3*). Measured data are shown as black crosses; the calculated profile is shown by a solid red line. The difference between the calculated and experimental data is shown as a blue profile. Magenta vertical bars represent the reflection positions for the phase.

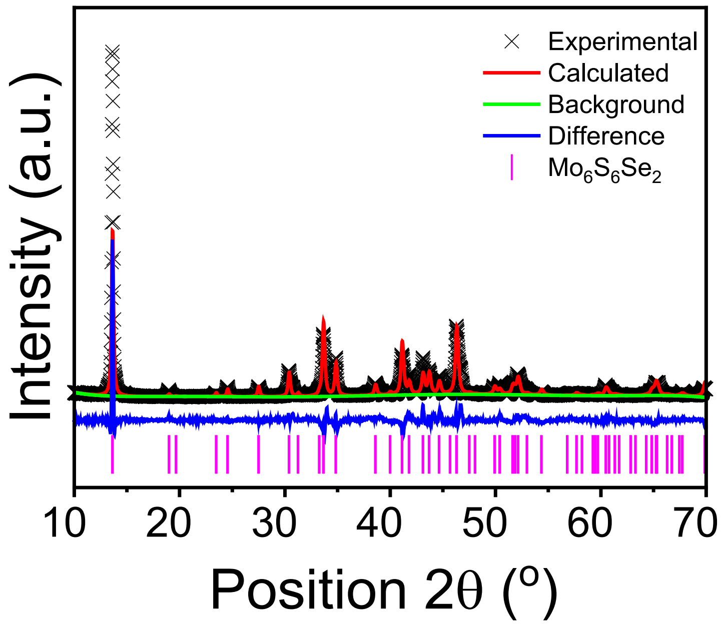

Figure S13. Rietveld refinement of the experimental PXRD profile (CuKα) for a sample with a nominal composition $Mo_6S_6Se_2$ against a structure model (Space group: R-*3*). Measured data are shown as black crosses; the calculated profile is shown by a solid red line. The difference between the calculated and experimental data is shown as a blue profile. Magenta vertical bars represent the reflection positions for the phase.

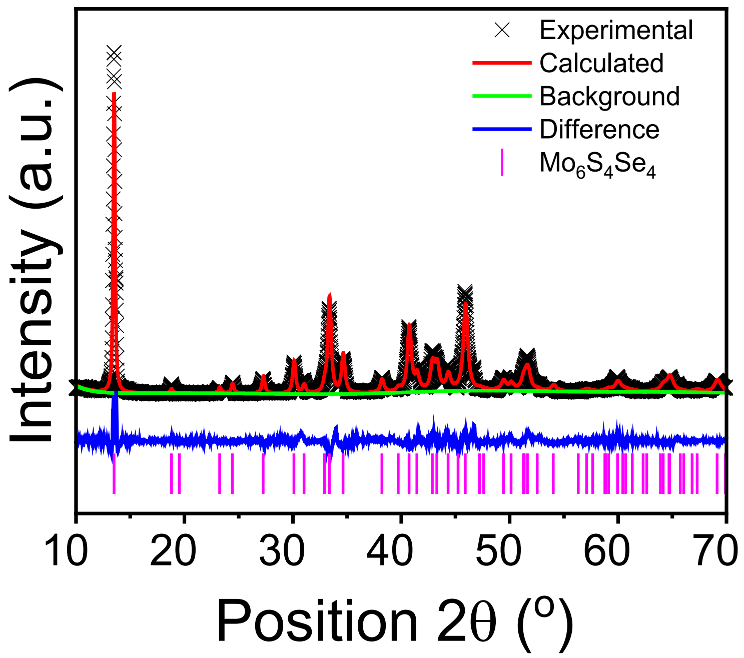

Figure S14. Rietveld refinement of the experimental PXRD profile (CuKα) for a sample with a nominal composition $Mo_6S_4Se_4$ against a structure model (Space group: R-*3*). Measured data are shown as black crosses; the calculated profile is shown by a solid red line. The difference between the calculated and experimental data is shown as a blue profile. Magenta vertical bars represent the reflection positions for the phase.

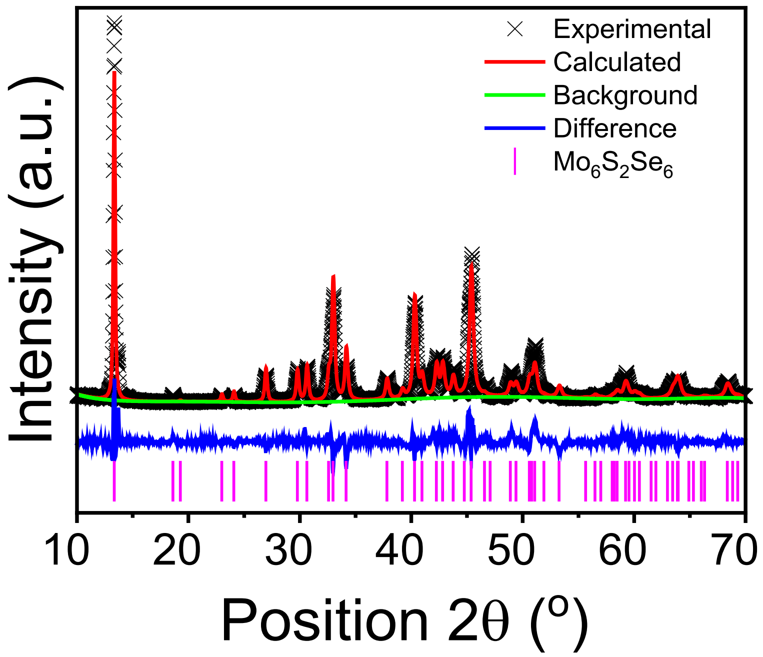

Figure S15. Rietveld refinement of the experimental PXRD profile (CuKα) for a sample with a nominal composition $Mo_6S_2Se_6$ against a structure model (Space group: R-*3*). Measured data are shown as black crosses; the calculated profile is shown by a solid red line. The difference between the calculated and experimental data is shown as a blue profile. Magenta vertical bars represent the reflection positions for the phase.

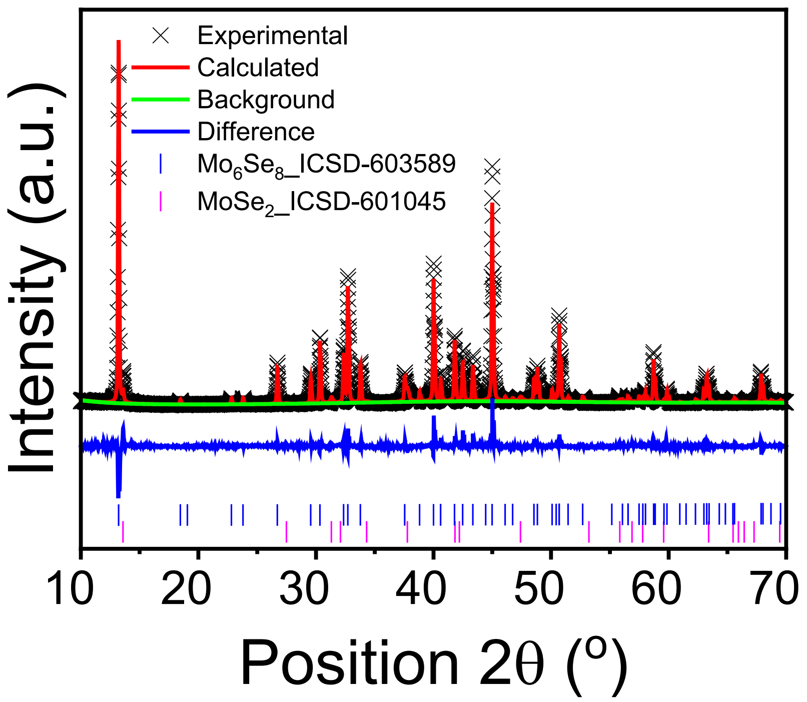

Figure S16. Rietveld refinement of the experimental PXRD profile (CuKα) for a sample with a nominal composition Mo_6Se_8 against a structure model (Space group: R-*3*). Measured data are shown as black crosses; the calculated profile is shown by a solid red line. The difference between the calculated and experimental data is shown as a blue profile. Magenta vertical bars represent the reflection positions for the phase.

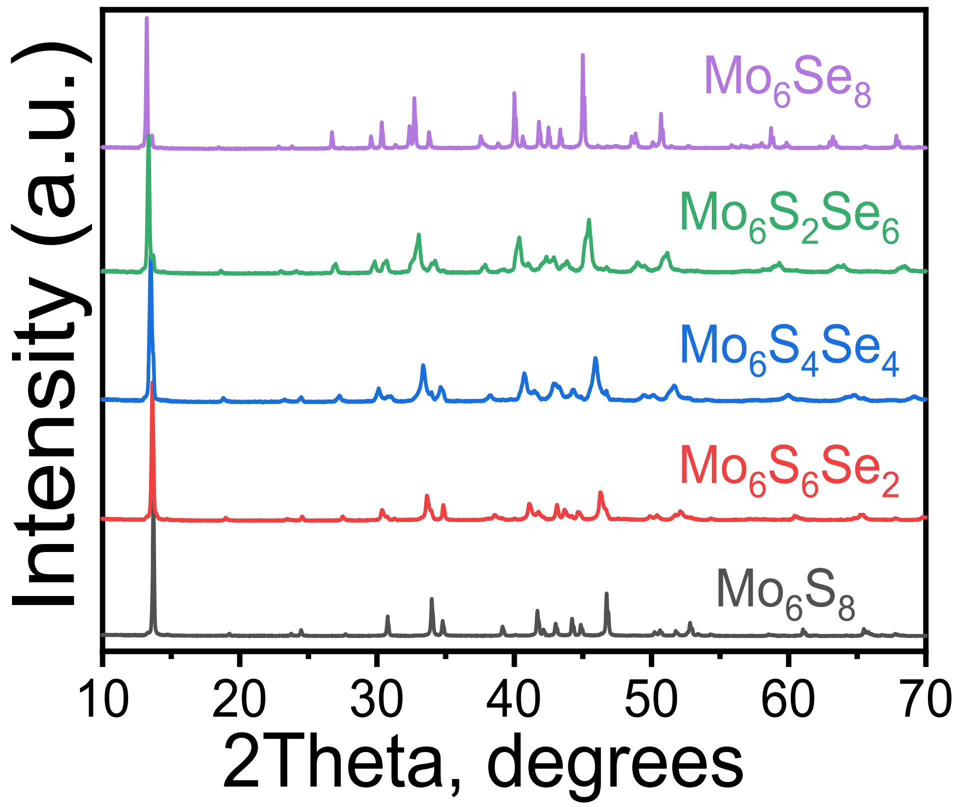

Figure S17. The experimental PXRD patterns of $Mo_6S_{8-x}Se_x$.

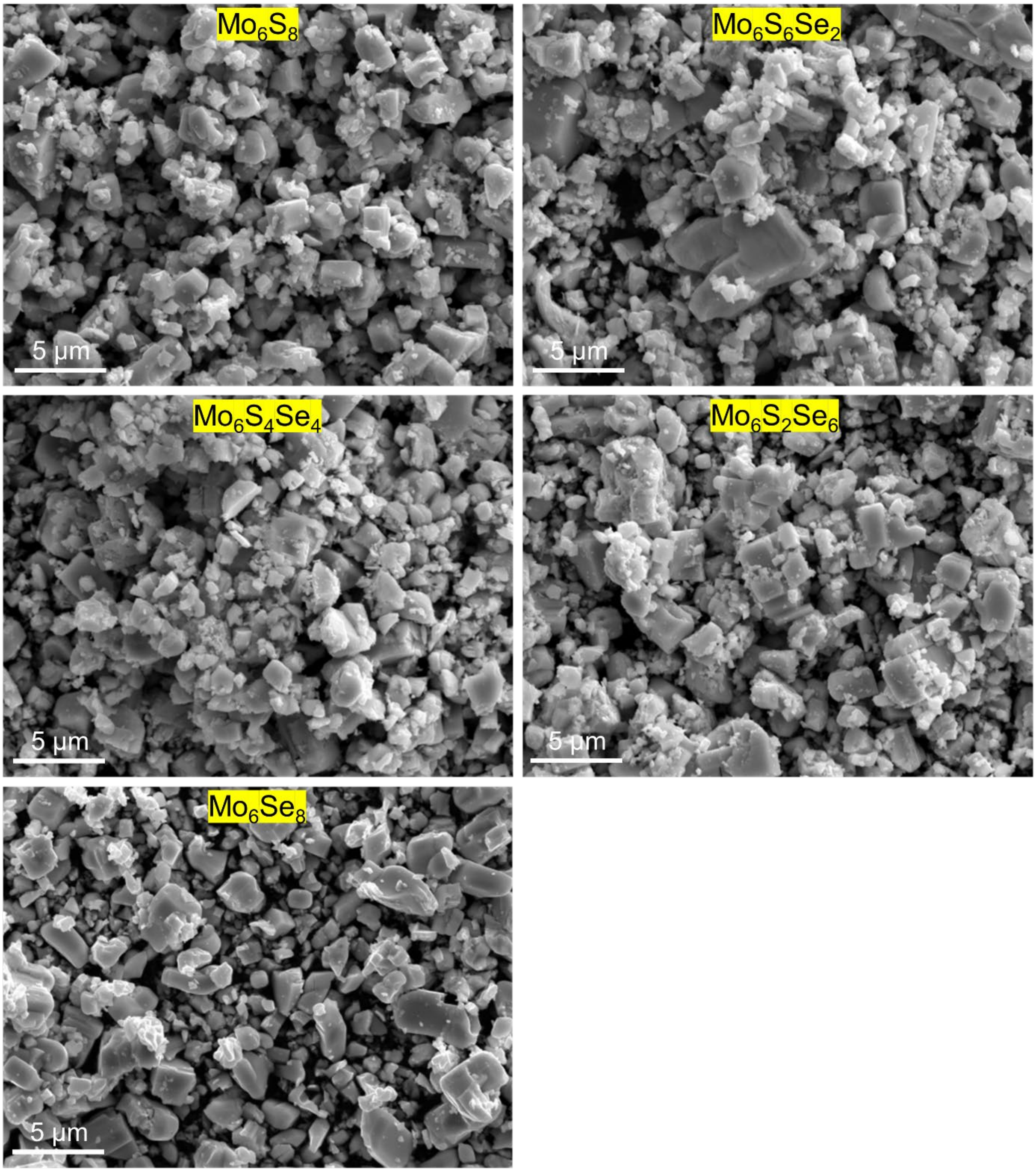

Figure S18. SEM images of Mo_6S_8, $Mo_6S_6Se_2$, $Mo_6S_4Se_4$, $Mo_6S_2Se_6$ and Mo_6Se_8.

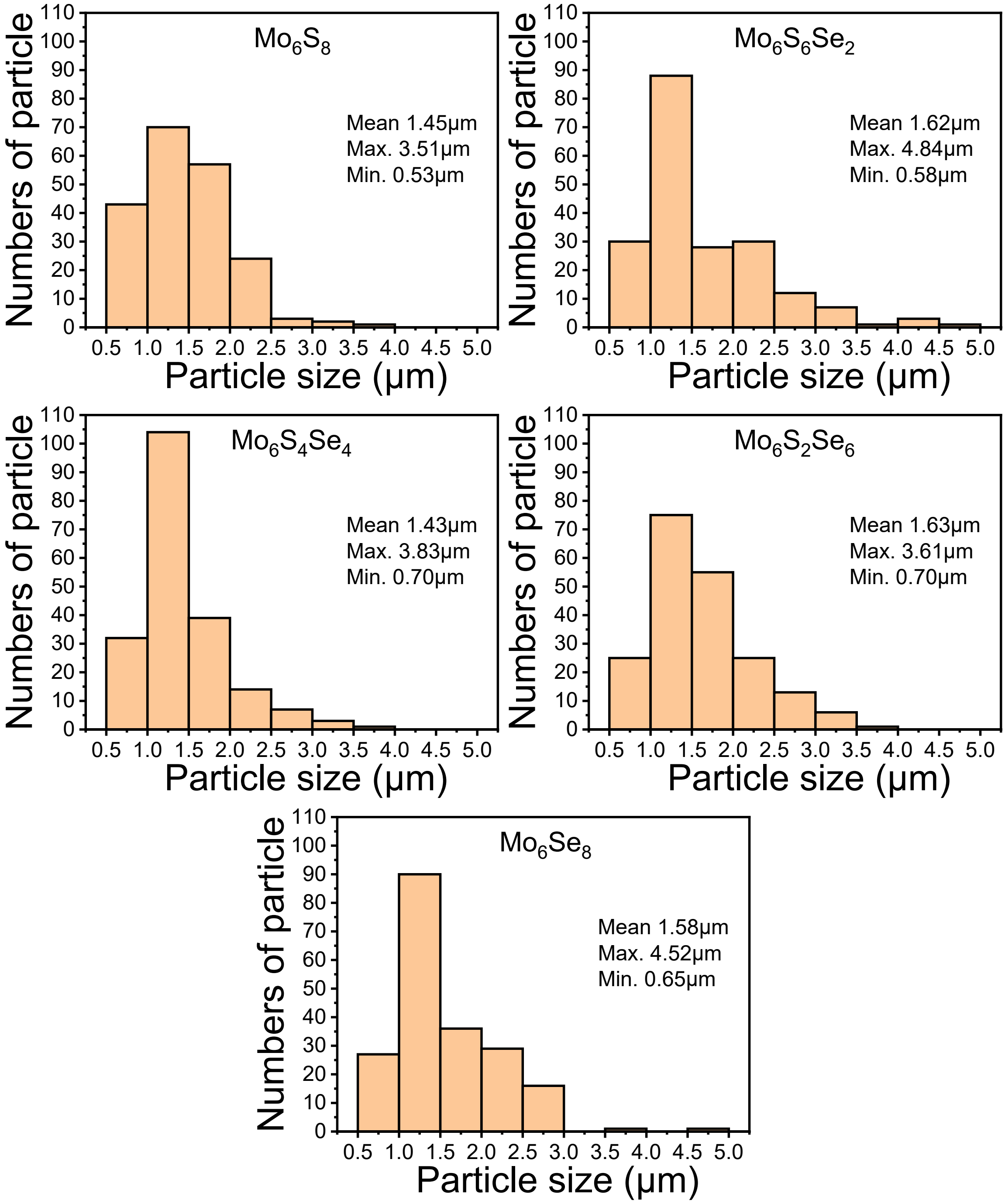

Figure S19. Particle size distribution in Mo_6S_8, $Mo_6S_6Se_2$, $Mo_6S_4Se_4$, $Mo_6S_2Se_6$ and Mo_6Se_8 samples evaluated from the corresponding images presented in Fig. S18 using Nanomeasurer software.

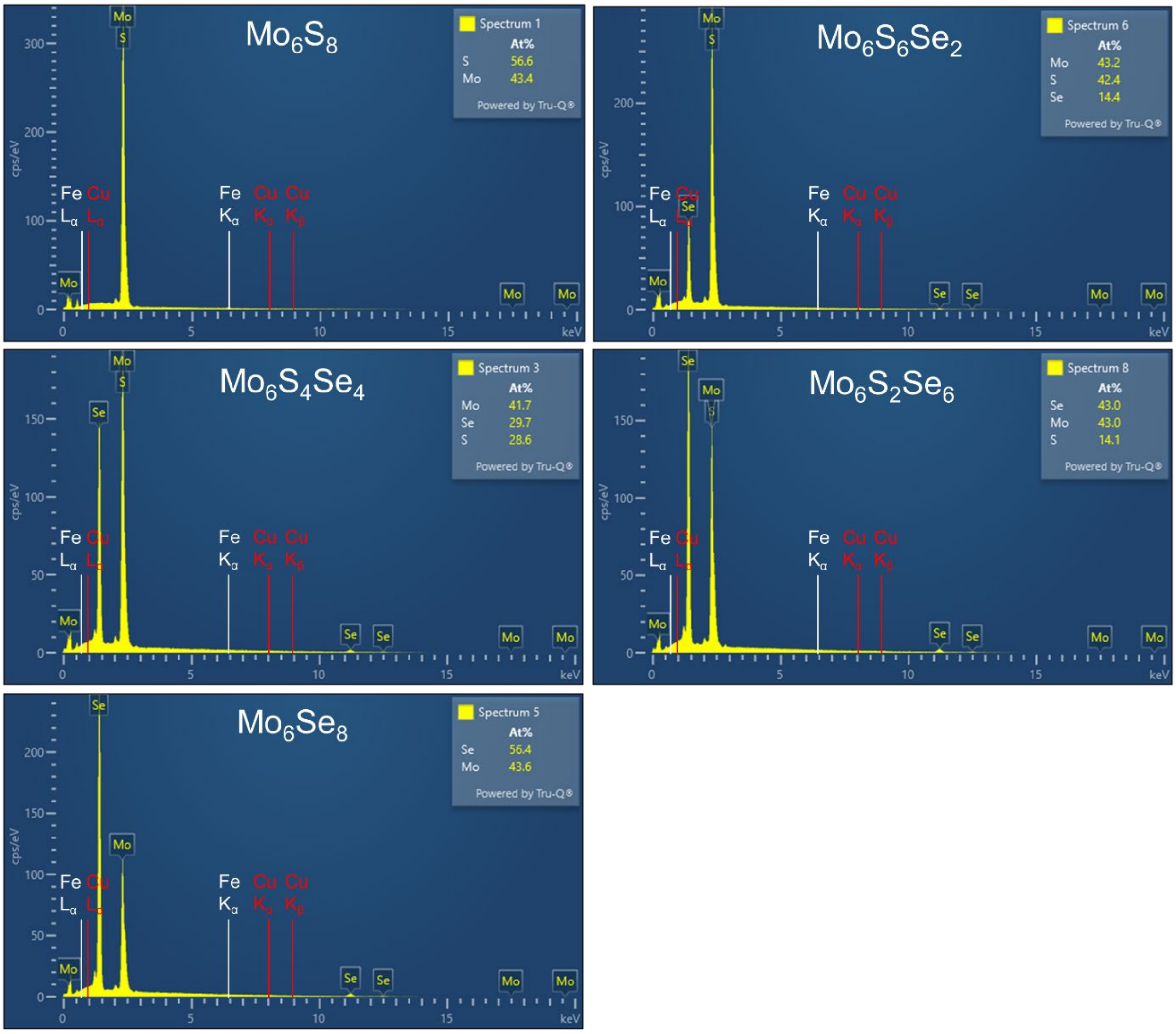

Figure S20. The representative EDX spectra of Mo_6S_8, $Mo_6S_6Se_2$, $Mo_6S_4Se_4$, $Mo_6S_2Se_6$ and Mo_6Se_8 samples with the expected positions of Cu and Fe peaks highlighted.

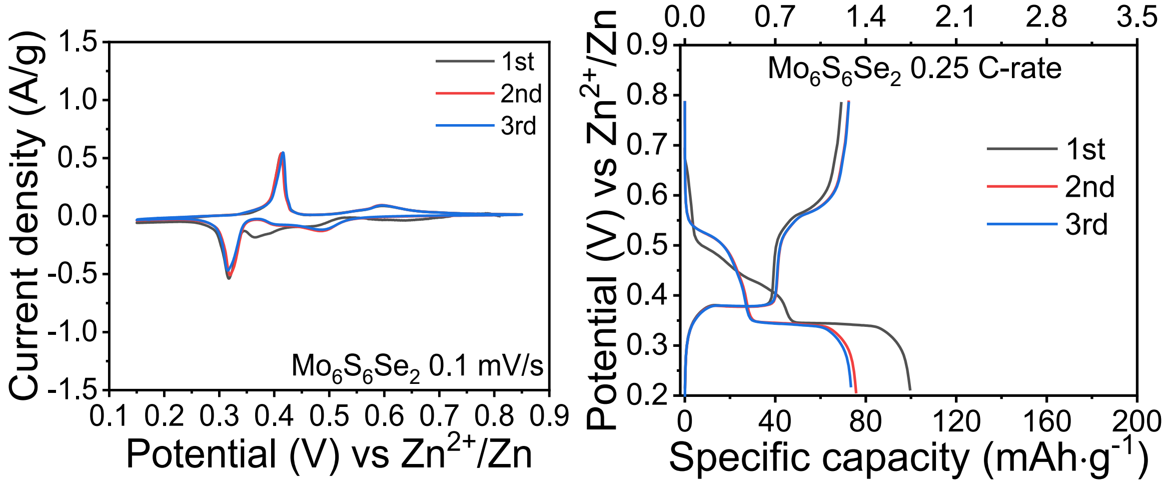

Figure S21. CV curves of $Mo_6S_6Se_2$ at 0.1 mV/s for 3 cycles started from OCV, and GCD profile of $Mo_6S_6Se_2$ at 0.25 C-rate, started from OCV.

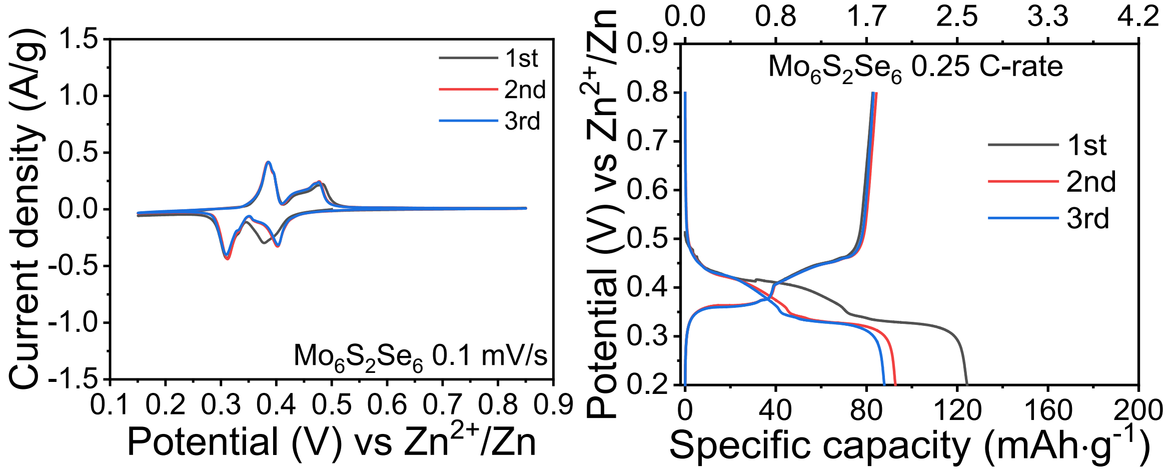

Figure S22. CV curves of $Mo_6S_2Se_6$ at 0.1 mV/s for 3 cycles started from OCV, and GCD profile of $Mo_6S_2Se_6$ at 0.25 C-rate, started from OCV.

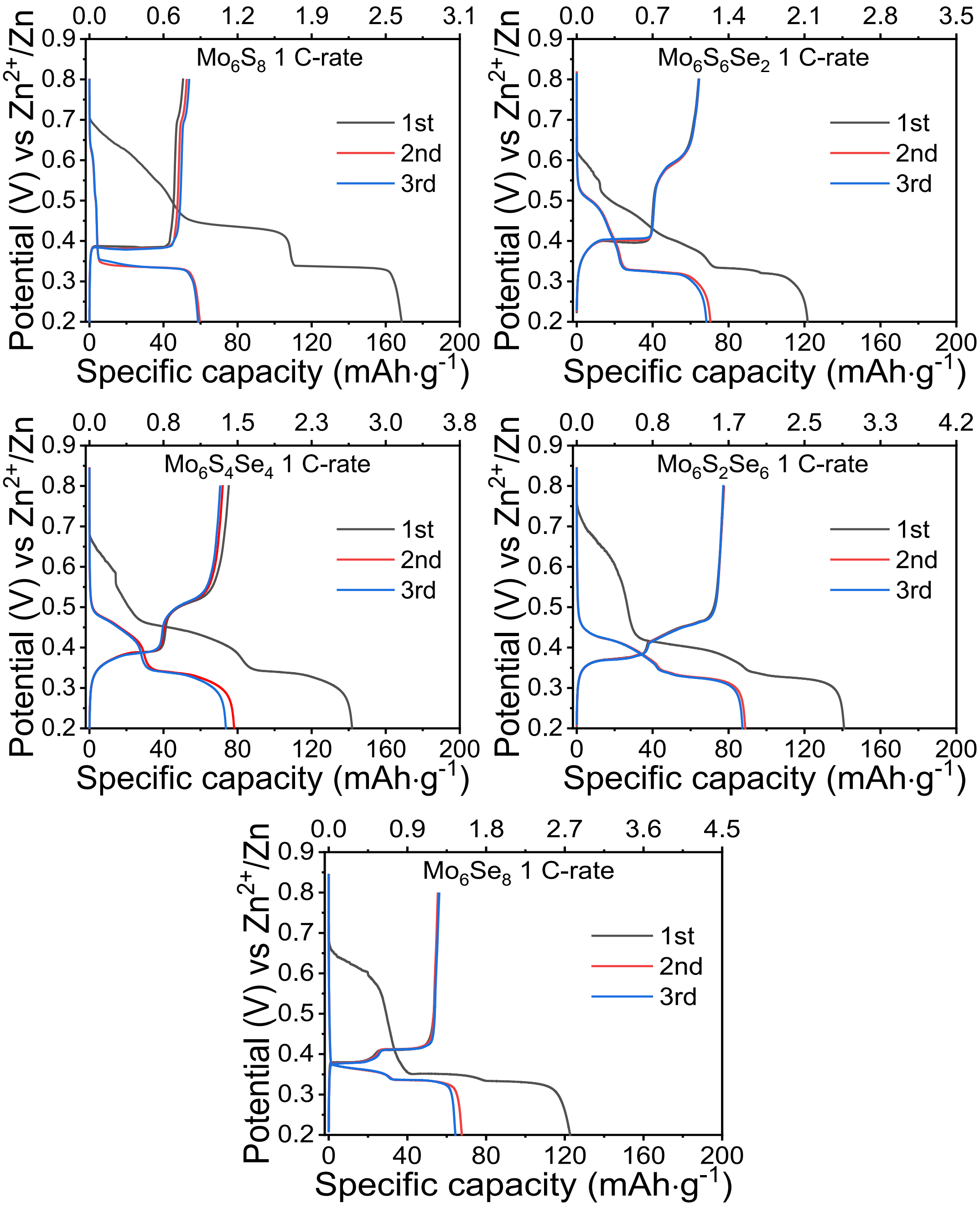

Figure S23. GCD profiles of Mo_6S_8, $Mo_6S_6Se_2$, $Mo_6S_4Se_4$, $Mo_6S_2Se_6$, and Mo_6Se_8 at 1 C-rate, started from OCV.

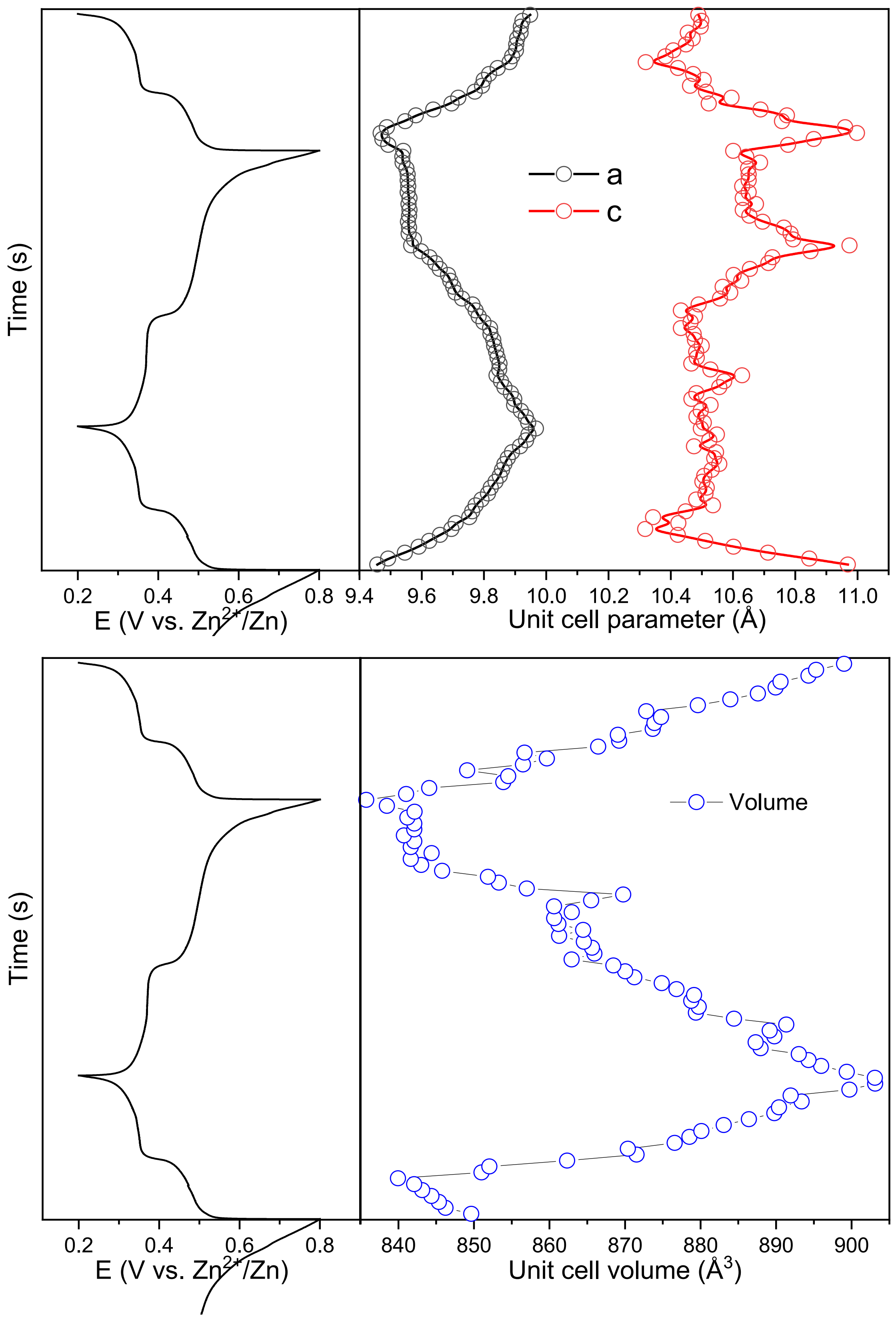

Figure S24. The variation of the $Mo_6S_4Se_4$ unit cell parameters upon Zn-ion insertion with respect to time and applied voltage.

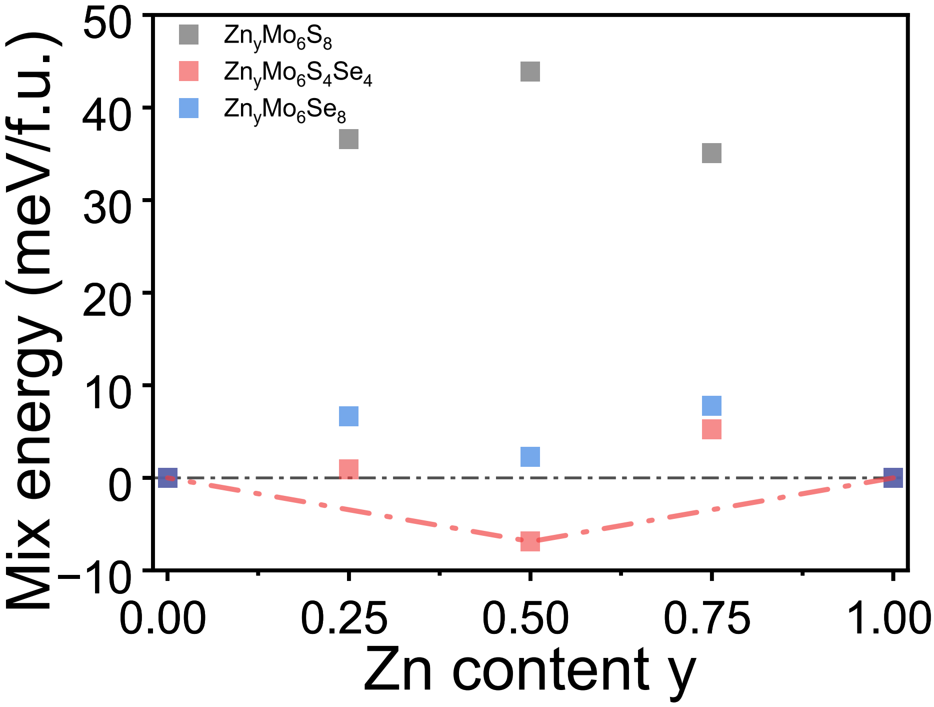

Figure S25. Mixing energies per formula unit (f.u.) of Mo_6S_8, $Mo_6S_4Se_4$ and Mo_6Se_8 and their change depending on Zn1 content. The energy hull relative to the phase separation into the end member of the series is shown in black dash line.

Supplementary Tables

Table S1. The number of the possible $Zn_yMo_6S_{8-x}Se_x$ (y = 0, 1 and 2; x = 0, 1, 2, 3, 4, 5, 6, 7 and 8) structure conformations of the reduced unit cell available for the DFT optimization.

	**y = 0**	**y = 1**	**y = 2**
**$Zn_yMo_6S_8$**	1	1	1
**$Zn_yMo_6S_7Se_1$**	8	8	8
**$Zn_yMo_6S_6Se_2$**	28	28	28
**$Zn_yMo_6S_5Se_3$**	56	56	56
**$Zn_yMo_6S_4Se_4$**	70	70	70
**$Zn_yMo_6S_3Se_5$**	56	56	56
**$Zn_yMo_6S_2Se_6$**	28	28	28
**$Zn_yMo_6S_1Se_7$**	8	8	8
**$Zn_yMo_6Se_8$**	1	1	1

Table S2. The optimized unit cell parameters of the most energetically stable $Mo_6S_{8-x}Se_x$, $ZnMo_6S_{8-x}Se_x$ and $Zn_2Mo_6S_{8-x}Se_x$ in the reduced cell configuration.

X in $Mo_6S_{8-x}Se_x$		*a*, Å	*b*, Å	*c*, Å	Alpha	Beta	Gamma	*V*, $Å^3$
0	Mo_6S_8	6.482	6.482	6.482	91.343	91.343	91.343	272.178
	$ZnMo_6S_8$	6.510	6.525	6.579	95.499	94.002	94.832	276.338
	$Zn_2Mo_6S_8$	6.642	6.593	6.658	95.330	95.147	96.706	286.762
1	$Mo_6S_7Se_1$	6.496	6.496	6.496	91.886	91.886	91.886	273.610
	$ZnMo_6S_7Se_1$	6.542	6.558	6.601	95.733	93.707	94.045	280.408
	$Zn_2Mo_6S_7Se_1$	6.681	6.615	6.683	95.610	94.597	96.398	290.849
2	$Mo_6S_6Se_2$	6.509	6.509	6.509	92.353	92.353	92.353	275.028
	$ZnMo_6S_6Se_2$	6.556	6.593	6.633	94.823	93.607	94.121	284.290
	$Zn_2Mo_6S_6Se_2$	6.701	6.662	6.718	94.905	94.778	95.935	295.854
3	$Mo_6S_5Se_3$	6.557	6.522	6.577	92.559	91.910	92.443	280.571
	$ZnMo_6S_5Se_3$	6.589	6.594	6.721	95.483	93.191	94.434	289.216
	$Zn_2Mo_6S_5Se_3$	6.699	6.667	6.811	95.833	93.981	95.918	300.028
4	$Mo_6S_4Se_4$	6.594	6.525	6.651	93.056	91.554	92.394	285.361
	$ZnMo_6S_4Se_4$	6.623	6.635	6.802	96.109	91.871	95.451	295.595
	$Zn_2Mo_6S_4Se_4$	6.716	6.689	6.920	96.287	93.142	96.206	306.454
5	Mo6S3Se5	6.627	6.604	6.700	92.675	91.623	92.401	292.521
	$ZnMo_6S_3Se_5$	6.636	6.714	6.816	95.814	92.051	95.928	300.166
	$Zn_2Mo_6S_3Se_5$	6.732	6.768	6.912	95.908	92.893	96.748	310.403
6	$Mo_6S_2Se_6$	6.706	6.628	6.685	92.422	92.157	91.263	296.629
	$ZnMo_6S_2Se_6$	6.793	6.671	6.803	95.973	93.665	94.310	304.973
	$Zn_2Mo_6S_2Se_6$	6.890	6.734	6.920	96.211	95.003	96.061	315.841
7	$Mo_6S_1Se_7$	6.683	6.707	6.758	92.190	91.313	91.621	302.477
	$ZnMo_6S_1Se_7$	6.814	6.755	6.802	95.987	93.477	95.031	309.439
	$Zn_2Mo_6S_1Se_7$	6.920	6.820	6.914	95.401	94.768	96.929	320.878
8	Mo_6Se_8	6.749	6.749	6.749	91.719	91.719	91.719	307.025
	$ZnMo_6Se_8$	6.828	6.852	6.850	95.233	94.647	95.073	316.610
	$Zn_2Mo_6Se_8$	6.941	6.885	6.940	94.865	95.276	96.753	326.304

Table S3. The summary of cell parameter of the $Mo_6S_{8-x}Se_x$ products determined by Rietveld refinement of the experimental profile against previously reported structural model for $Mo_6S_{8-x}Se_x$. The standard deviations are given in parentheses.

	a, Å	*c*, Å	*V*, Å³	Reference
Mo_6S_8	9.1793(1)	10.8715(1)	793.314(9)	This work
Mo_6S_8	9.1910	10.8785	795.84	[4]
$Mo_6S_6Se_2$	9.3175(7)	10.8498(7)	815.754(10)	This work
$Mo_6S_4Se_4$	9.4006(8)	10.9012(8)	834.302(10)	This work
$Mo_6S_4Se_4$	9.364	11.006	835.76	[5]
$Mo_6S_2Se_6$	9.4889(7)	11.0488(8)	861.556(9)	This work
Mo_6Se_8	9.5484(1)	11.1582(1)	881.022(6)	This work
Mo_6Se_8	9.537	11.239	885.28	[6]

Table S4. The atomic ration of $Mo_6S_{8-x}Se_x$ solid solutions based on the EDXS

	Mo at. %		S at. %		Se at. %	
	Expected	Experiment	Expected	Experiment	Expected	Experiment
Mo_6S_8	42.9	43.4	57.1	56.6	0	0
$Mo_6S_6Se_2$	42.9	43.2	42.9	42.4	14.3	14.4
$Mo_6S_4Se_4$	42.9	41.7	28.6	28.6	28.6	29.7
$Mo_6S_6Se_2$	42.9	43.0	14.3	14.1	42.9	43.0
Mo_6Se_8	42.9	43.6	0	0	57.1	56.4

Table S5. Specific discharge capacity reached at the 3rd cycle by a given sample depending on C-rate.

	**Specific discharge capacity (mAh g^{-1})**				
**C-rate**	**Mo_6S_8**	**$Mo_6S_6Se_2$**	**$Mo_6S_4Se_4$**	**$Mo_6S_2Se_6$**	**Mo_6Se_8**
**0.25**	68.6	73.4	81.9	87.8	82.8
**1**	58.6	68.3	73.6	87.3	64.4

Table S6. The calculated unit cell parameters of $Zn_2Mo_6Se_8$ based on the limited data within the measured range of 2Theta for *in operando* data presented in Fig. 4.

a (Å)	c (Å)	V (Å^3)	Reference
10.076	10.936	961.6	This work
10.258	10.451	952.4	[7]
10.053	10.824	947.2	[8]

REFERENCES

(1) Umarji, A.; Rao, G. S.; Janawadkar, M. P.; Radhakrishnan, T. S. Metal atom incorporation studies on $A_xMo_6S_8$ Chevrel phases. *J. Phys. Chem. Solids* **1980**, 41, 421–429.

(2) Johnson, D. C.; Tarascon, J. M.; Sienko, M. J. Chalcogen ordering on special-position sites in ternary molybdenum chalcogenides. *Inorg. Chem.* **1983**, 22, 3773–3776.

(3) Helmbrecht, K.; Euchner, H.; Groß, A. Revisiting the chevrel phase: Impact of dispersion corrections on the properties of Mo_6S_8 for cathode applications. *Batteries & Supercaps* **2022**, 5, e202200002.

(4) Chae, M. S.; Heo, J. W.; Lim, S. C.; Hong, S. T. Electrochemical zinc-ion intercalation properties and crystal structures of $ZnMo_6S_8$ and $Zn_2Mo_6S_8$ chevrel phases in aqueous electrolytes. *Inorg. Chem.* **2016**, 55, 3294–3301.

(5) Tarascon, J. M.; DiSalvo, F. J.; Waszczak, J. V.; Hull Jr, G. W. Synthesis and peculiar properties of $InMo_6S_{8-x}Se_x$, $TlMo_6S_{8-x}Se_x$, and $Hg_yMo_6S_{8-x}Se_x$. *Phys. Rev. B* **1985**, 31, 1012.

(6) Tarascon, J. M.; DiSalvo, F. J.; Murphy, D. W.; Hull, G.; Waszczak, J. V. New superconducting ternary molybdenum chalcogenides $InMo_6Se_8$, $T1Mo_6S_8$, and $T1Mo_6Se_8$. *Phys. Rev. B* **1984**, 29, 172.

(7) Jadhav A.L.; Juran, T.R.; Kim, M.A.; Bruck, A.M.; Hawkins, B.E.; Gallaway, J.W.; Smeu, M.; Messinger, R.J. Reversible electrochemical anionic redox in rechargeable multivalent-ion batteries. *J. Am. Chem. Soc.* **2023**, 145, 15816–15826.

(8) Gocke E.; Schramm W.; Dolscheid P.; R. Schöllhorn. Molybdenum cluster chalcogenides Mo_6X_8: Electrochemical intercalation of closed shell ions Zn^{2+}, Cd^{2+}, and Na. *J. Solid State Chem.* **1987**, 70, 71–81.
